# c-Jun N-terminal kinase 1 (JNK1) modulates oligodendrocyte progenitor cell architecture, proliferation and myelination

**DOI:** 10.1038/s41598-021-86673-6

**Published:** 2021-03-31

**Authors:** Martina Lorenzati, Enrica Boda, Roberta Parolisi, Martino Bonato, Tiziana Borsello, Thomas Herdegen, Annalisa Buffo, Alessandro Vercelli

**Affiliations:** 1 Department of Neuroscience Rita Levi-Montalcini, University of Turin, Turin, Italy; 2 Neuroscience Institute Cavalieri Ottolenghi (NICO), University of Turin, Orbassano (Turin), Italy; 3 Istituto di Ricerche Farmacologiche Mario Negri-IRCCS, Milan, Italy; 4 Department of Pharmacological and Biomolecular Sciences, University of Milan, Milan, Italy; 5 Institute of Experimental and Clinical Pharmacology, University Hospital Schleswig-Holstein, Kiel, Germany

**Keywords:** Oligodendrocyte, Glial biology

## Abstract

During Central Nervous System ontogenesis, myelinating oligodendrocytes (OLs) arise from highly ramified and proliferative precursors called oligodendrocyte progenitor cells (OPCs). OPC architecture, proliferation and oligodendro-/myelino-genesis are finely regulated by the interplay of cell-intrinsic and extrinsic factors. A variety of extrinsic cues converge on the extracellular signal-regulated kinase/mitogen activated protein kinase (ERK/MAPK) pathway. Here we found that the germinal ablation of the MAPK c-Jun N-Terminal Kinase isoform 1 (JNK1) results in a significant reduction of myelin in the cerebral cortex and corpus callosum at both postnatal and adult stages. Myelin alterations are accompanied by higher OPC density and proliferation during the first weeks of life, consistent with a transient alteration of mechanisms regulating OPC self-renewal and differentiation. JNK1 KO OPCs also show smaller occupancy territories and a less complex branching architecture in vivo. Notably, these latter phenotypes are recapitulated in pure cultures of JNK1 KO OPCs and of WT OPCs treated with the JNK inhibitor D-JNKI-1. Moreover, JNK1 KO and WT D-JNKI-1 treated OLs, while not showing overt alterations of differentiation in vitro, display a reduced surface compared to controls. Our results unveil a novel player in the complex regulation of OPC biology, on the one hand showing that JNK1 ablation cell-autonomously determines alterations of OPC proliferation and branching architecture and, on the other hand, suggesting that JNK1 signaling in OLs participates in myelination in vivo.

## Introduction

During CNS development, myelin is produced by mature oligodendrocytes (OLs), whose cell processes spirally ensheathe discrete axon segments called internodes^1^. This arrangement assures a high-speed action potential conduction along neuronal axons^2^ and is instrumental to the achievement of impulse discharge synchrony among neurons^3^.

Myelinating OLs originate from parenchymal precursors expressing the neuron-glia antigen 2 (NG2) chondroitin sulphate proteoglycan and the platelet derived growth factor receptor alpha (PDGFRα), commonly referred to as oligodendrocyte progenitor cells (OPCs)^4^. These cells initially amplify and colonize the entire CNS and maintain the proliferating ability even during adult life^5^. Nevertheless, OPC overgrowth is constantly prevented by OPC-to-OPC repulsion. OPCs have a complex ramified morphology with multiple and dynamic processes that continuously scan the environment and repel each other through contact-mediated inhibition^6, 7^. This assures the achievement of a “grid-like” homogenous cell distribution, where individual OPCs occupy non-overlapping domains. The progression of OPCs towards myelination is a multistep process that implies profound molecular changes and remarkable morphological rearrangement^8, 9^. Both a cell-intrinsic program and environmental factors participate in the regulation of OPC architecture, proliferation and oligodendro-/myelino-genesis. Extrinsic signals include contact-mediated and soluble factors provided by neurons and glial cells, including OPC themselves^8, 10^. In spite of their origin, a variety of these cues converge on the extracellular signal regulated kinase/mitogen activated protein kinase (ERK/MAPK) pathway in OPCs/OLs^11, 12^, although the identity and the specific role of the signal transduction players active in oligodendroglia at distinct functional phases remain poorly understood.

Among MAPKs, the c-Jun N-terminal kinases (JNKs) include three isoforms—JNK1, JNK2 and JNK3. While JNK1 and JNK2 are expressed ubiquitously, JNK3 expression is restricted to a few regions, including the brain^13^. Despite this, in this latter region JNK1 seems to have a predominant role, as JNK1 KO mouse brain shows developmental abnormalities^14^, including alterations in neuronal specification^15^, microtubule integrity^16, 17^, cell migration^18^, dendritic and spine architecture^19, 20^ and developmental apoptosis^21^. Oligodendroglial cells are known to express all the three JNK isoforms^22^. JNK3 has been consistently reported as a regulator of OPC/OL sensitivity to apoptosis^23–25^ whereas the roles of JNK1 and JNK2 isoforms^26^ have not been investigated so far. Yet, the multifaceted contribution of JNK1 to neuronal development suggests this kinase may also exert multiple functions in non-neuronal cells, including oligodendroglia.

Here we found that the germinal ablation of JNK1 (JNK1 KO) results in a reduced expression of myelin proteins in the cerebral cortex and corpus callosum (CC) at both postnatal and adult stages. This occurred in spite of similar axonal densities in WT and JNK1 KO mouse cortex and CC. In these regions, OPC density and proliferation rate were higher during the first two weeks of life, consistent with the idea of alterations in mechanisms regulating oligodendroglial proliferation and differentiation. Of note, JNK1 KO OPCs also showed smaller occupancy territories and a less complex branching architecture in vivo. These phenotypes were all recapitulated in pure cultures of JNK1 KO OPCs and WT OPCs treated with the JNK-inhibitor D-JNKI-1, indicating a cell-autonomous role for JNK1 in the regulation of OPC amplification and morphology. On the contrary, mutant and D-JNKI-1 treated OPCs did not show overt alterations of differentiation in vitro. However, they displayed a decreased surface extension suggesting that cell-autonomous factors may participate in cortical/CC hypomyelination in JNK1 KO mice. These data point to JNK1 as a novel player in the complex regulatory network of oligodendroglia functions and myelination.

## Results

### JNK1 KO mice display myelin abnormalities

**Figure 1:**
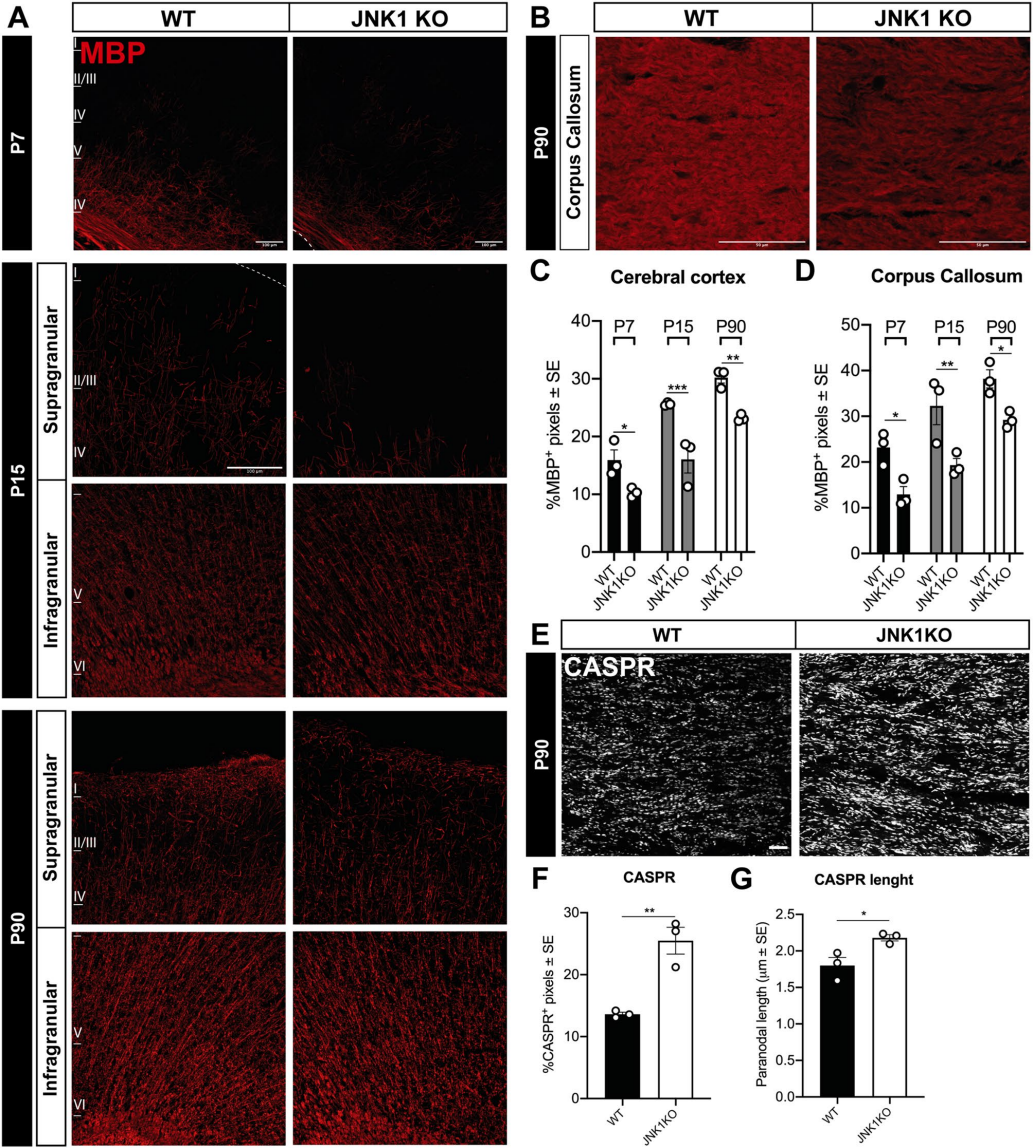
Myelin alterations in JNK1 KO mouse cortex and CC. (**A**) Myelin distribution (red) in the cortex of P7, P15 and P90 WT vs JNK1KO mice. At P7 images illustrate the deep layers of the somatosensory cortex, at P15 and P90 images illustrate separately infragranular and supragranular cortical layers. (**B**) Representative images of MBP (red) distribution in P90 WT vs JNK1KO mice CC. (**C**, **D**) Quantification of the percentage of MBP^+^ pixels in the cortex (**C**) and in the CC (**D**) of P7, P15 and P90 WT vs JNK1KO mice. (**E**) Distribution of CASPR^+^ paranodes in the CC of P90 WT vs JNK1KO mice, and (**F**) quantification of the percentage of CASPR^+^ pixels. (**G**) Quantification of the length of CC CASPR^+^ paranodes in P90 WT vs JNK1KO mice. Scale bars: 100 μm in (**A**) and (**B**), 10 μm in (**E**). *WT* wild type, *P* postnatal day, *MBP* Myelin Basic Protein, *CASPR* contactin-associated protein. *P < 0.05; **P < 0.01; ***P < 0.001.

**Figure 2:**
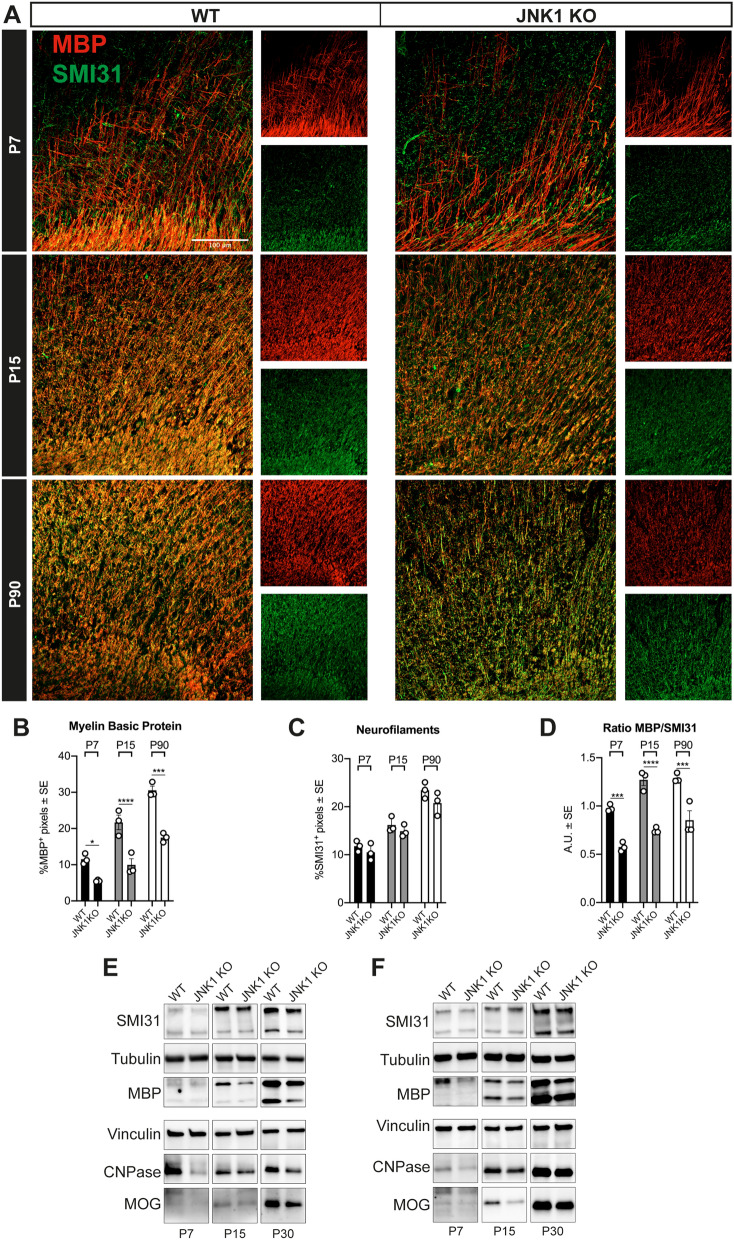
Myelin alterations in JNK1 KO are not related to axonal abundance. (**A**) MBP^+^ (red) and SMI31 neurofilaments (green) expression in the cortex and CC of P7, P15 and P90 WT vs JNK1KO mice. At P7 images illustrate the deep layers of the motor cortex, nearby cingulum bundle. Quantification of the percentage of MBP^+^ (**B**), and SMI31 (**C**) pixels in the cortex and in CC of WT vs JNK1KO mice, and their ratio (**D)**. (**E**) Western blots of P7, P15 and P30 WT and JNK1KO cortices and (**F**) corpora callosa. Full length blots are presented in Suppl. Fig. 5 (*A*—cortices, *B—*corpora callosa). Scale bars: 100 μm. *WT* wild type, *P* postnatal day, *A.U.* arbitrary units, *MBP* Myelin Basic Protein, *SMI31* neurofilaments, *CNPase* 2′,3′-Cyclic-Nucleotide 3′-phosphodiesterase, *MOG* Myelin Oligodendrocyte Glycoprotein. *P < 0.05; **P < 0.01; ***P < 0.001; ****P < 0.0001.

In order to address the impact of JNK1 ablation on oligodendroglia, we firstly examined the expression of the myelin marker MBP in the cerebral cortex. We found that JNK1 KO mice display a lower expression of MBP, both in infragranular and supragranular layers of the somatosensory cortex *(*Fig. 1A,C and Suppl. Fig. 1A,B) and in the CC (Fig. 1B,D). This defect was found at postnatal ages (P7 and P15) and persisted at adult stages (P90). Myelin abnormalities, not only restricted to MBP expression, were also confirmed by observation of WT and JNK1 KO Gallyas-stained sagittal sections (Suppl. Fig. 1C). Former studies on JNK1 KO revealed some extent of axonal degeneration^17^. Thus, we asked whether the observed reduction of MBP and myelin reflected the axonal regressive events. Indeed, the ratio of MBP/healthy axons (as detected by labelling of SMI31, a phosphorylated epitope of neurofilament H, a major component of the axonal cytoskeleton (Fig. 2A; Yandamuri et al.^27^)) appeared to be reduced in JNK1 KO cortices and CC compared to WT, and the axon densities did not display a major decrease in mutant mice within the analyzed time window (Fig. 2A–D). These histological results were in line with western blotting (WB) analyses, which confirmed a reduction in the amount of other myelin-associated proteins, such as 2′,3′-Cyclic-Nucleotide 3′-phosphodiesterase (CNPase) and Myelin Oligodendrocyte Glycoprotein (MOG) (Fig. 2E,F and Suppl. Fig. 2A,B). On the whole, these results suggest hypomyelination in JNK1 KO mice. Also, myelin alterations were not merely attributable to a decrease of mature OLs in JNK1 KO mice as the densities of CC1^+^ OLs were overall comparable to those of WTs in both cerebral cortex and CC (Suppl. Fig. 3A,B).

**Figure 3:**
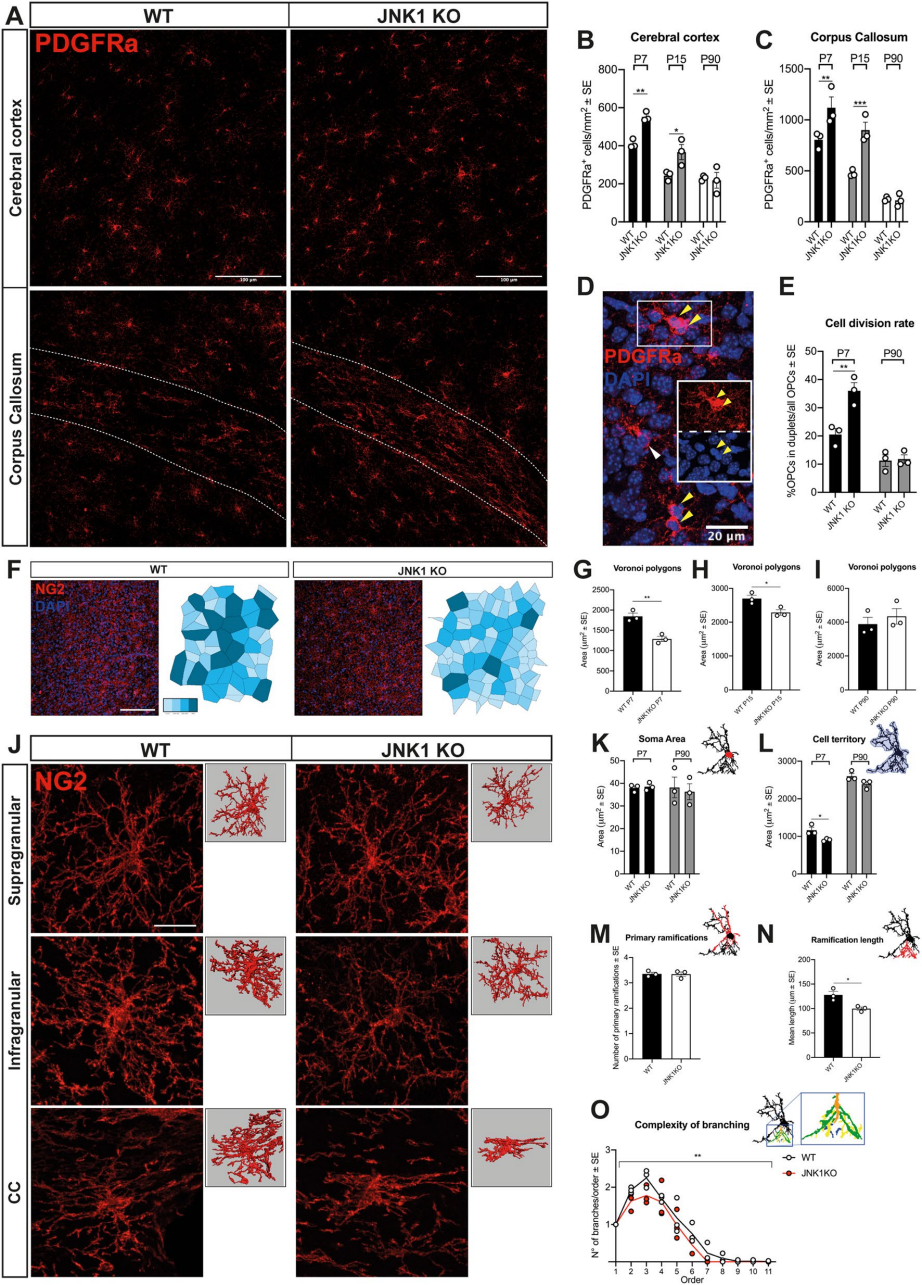
JNK1 loss increases OPC proliferation and alters OPC territory and architecture in vivo*.* (**A**) Distribution of PDGFRα^+^ OPCs (red) in WT vs JNK1KO cortex and CC (area between dotted lines) at P7. (**B, C**) Quantification of PDGFRα^+^ OPCs in the dorsal cortex (**B**) and CC (**C**) at P7, P15 and P90. (**D**) Representative image of a PDGFRα^+^ (red) OPC duplet (i.e. juxtaposed sister cells just exiting cytokinesis and showing juxtaposed and symmetrical cell somata with decondensed grainy DAPI staining, yellow arrows) in the cortical grey matter. White arrow points to an individual non-newly generated OPC. DAPI (blue) counterstains cell nuclei. (**E**) Quantification of the fraction of PDGFRα^+^ OPCs in duplets over the OPC population (as a measure of OPC division rate) at P7 and P90. (**F**) Distribution of NG2^+^ OPCs (red) and construction of related Voronoi polygons in the cerebral cortex of P7 WT vs JNK1KO mice. Each Voronoi polygon is colour-coded according to the size of the area. DAPI (blue) counterstains cell nuclei. (**G**,** H**,** I**) Areas covered by the Voronoi polygons in (**G**) P7, (**H**) P15 and (**I**) P90 WT vs JNK1KO mice. (**J-O**) Morphology and morphometry of WT vs JNK1KO NG2^+^ OPCs. (**J**) Representative images of P7 WT vs JNK1 KO NG2^+^ OPCs (red) in supragranular and infragranular layers of the cortex and in the CC, with related Imaris reconstruction (panels on the right). (**K**, **L**) Quantification of the soma area (**K**) and of the territory occupied by OPC ramifications (**L**) in P7 and P90 WT vs JNK1 KO mice. (**M–O**) Quantification, through Neurolucida reconstruction, of the number of primary ramifications (**M**), mean length (**N)** and complexity (**O**) of the ramifications in P90 WT vs JNK1 KO NG2^+^ OPCs. Asterisks in (**O**): Two-way ANOVA, main effect of genotype. Scale bars: 100 μm in (**A**), 100 μm in (**F**), 20 μm in (**L**). *WT* wild type, *P* postnatal day, *PDGFRα* platelet-derived growth factor receptor A, *NG2* neural/glial antigen 2. *P < 0.05; **P < 0.01; ***P < 0.001; ****P < 0.0001.

To assess whether changes in myelin levels in mutants were accompanied by alterations of the axo-myelinic arrangement, we examined the nodal/paranodal region by immunostaining against the paranodal protein CASPR^28, 29^. Quantification of CASPR^+^ segments in the CC of adult brains revealed a significant staining increase in JNK1 KO samples (Fig. 1E,F). Moreover, analyses of CASPR^+^ node/paranode length showed a 17% increase in CASPR^+^ segment length in mutants as compared to control mice (Fig. 1G). Of note, this latter feature is frequently found in hypo-/dys-myelinating conditions^30, 31^, corroborating the idea of myelin alterations in JNK1 KO mice cortex and CC.

### JNK1 KO cortical OPCs display enhanced proliferation early after birth and morphological alterations

As a second step, we expanded the investigation on OPCs and assessed their density, proliferation rate and apoptosis at different survival times (P7, P15 and P90). We found a significant increase (about 34%) in the density of PDGFRα^+^ OPCs at P7 and P15 in KO mice compared to WT, both in cerebral cortex and CC (Fig. 3A–C, representative images at P7) with no changes in cell distribution throughout the cortical layers (Suppl. Fig. 3C). Since the presence of a higher number of OPCs in the JNK1 KO cortex could result from either higher cell proliferation or decreased apoptosis (or a combination of the two), we counted PDGFRα^+^ duplets as a measure of proliferative OPCs^5, 32^. At P7, the fraction of OPCs in duplets in JNK1 KO cortices was almost twofold higher than in WT, revealing that mutant OPCs have a higher proliferation rate than WT cells *(*Fig. 3D,E). Yet, the normal density (Fig. 3B,C) as well as the OPC proliferative fraction (Fig. 3E) of JNK1 KO OPCs appeared restored at adult stages (P90), suggesting a higher susceptibility of young OPCs to JNK1-dependent regulatory mechanisms. These results were also confirmed by analyses of NG2^+^/Ki67^+^ OPCs (Suppl. Fig. 3D).

Conversely, when we examined NG2^+^ OPCs expressing activated caspase 3 (cCASP3) to detect ongoing apoptosis, co-expressing OPCs were barely detected in both WT and JNK1-KO mice *(not shown).* Similar results were also obtained by TUNEL staining (Suppl. Fig. 3E). These data point to JNK1 participation in the regulation of OPC proliferation, at least in a developmental time window.

Based on increased OPC density, we hypothesized that the territory occupied by each cell could also be altered in JNK1 KO cortices. This hypothesis was initially tested by the analysis of the Voronoi polygons, a tool to analyze the spatial distribution of cells^33–35^. Voronoi analysis suggested that, during early developmental stages (P7-P15), JNK1 KO OPCs occupied a less extended area than WT cells (Fig. 3F–I). To further corroborate these data and better understand the underlying cellular features, we performed morphometric analyses of both OPC somata and branches (Fig. 3J–L). Analyses at early and adult stages showed that OPC soma areas did not differ between WT and KO cells (Fig. 3K). However, in agreement with the Voronoi results, OPC territory (i.e. the area occupied by the entire OPC extension, including cell ramification) was significantly smaller in JNK1 KO than in WT (Fig. 3L). Yet, this decrease was no longer appreciated at adult stages (Fig. 3L). Nevertheless, at P90, JNK1 KO OPCs displayed a shorter total length of ramifications with no changes in the number of primary ramifications (Fig. 3M,N) and a lower ratio of the number of branches over branch order (Fig. 3O). Thus, mutant OPC processes appeared less complex and overall less extended compared to the WT ones.

Taken together, these data indicate that JNK1 may play a role in the OPC proliferation and in the regulation of OPC branching architecture.

### Cultured JNK1 KO OPCs reproduce proliferative and morphological alterations found in vivo

In order to disentangle whether JNK1 KO OPC alterations in vivo depended on other cell types or could be explained cell autonomously, we performed cultures of MACS-isolated OPCs derived from P0 WT or JNK1 KO mice and examined cell proliferation, apoptosis and morphology.

At first, we tested the occurrence of possible dysregulated expression of the other JNK isoforms, potentially accounting for compensatory mechanisms or functional alterations. However, levels of JNK2 and JNK3 expression in acutely isolated JNK1 KO cells, as tested by qRT-PCR, were comparable to those of WT cells (Suppl. Fig. 4C–E), thus confirming that we were assessing the consequence of JNK1 abrogation.

**Figure 4:**
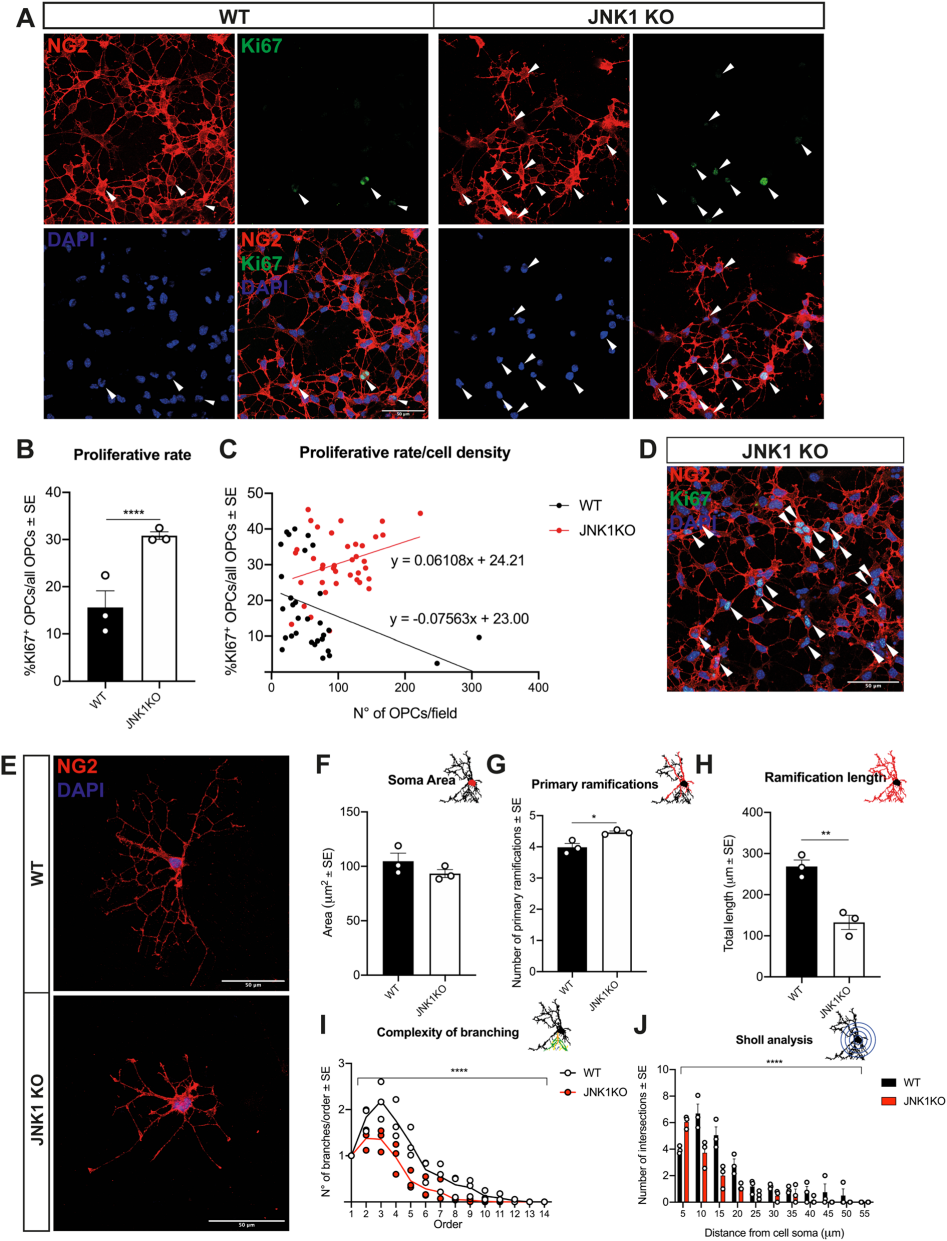
JNK1KO OPCs show higher proliferation and less complex ramifications in vitro*.* (**A**) Representative images of MACS-sorted cultured WT or JNK1 KO NG2^+^ (red) OPCs. Ki67^+^ proliferating cells (green) are indicated by white arrows. DAPI (blue) counterstains cell nuclei. (**B**) Quantification of WT vs JNK1 KO MACS-sorted proliferating OPCs. In (**C**) the proliferative fraction (Ki67^+^ OPCs over all OPCs) is plotted as a function of the number of OPCs in each analyzed field. The result of this analysis was also confirmed excluding the WT leverage points. (**D**) Representative image of MACS-sorted cultured JNK1 KO OPCs in high cell density. (**E**) Representative images of WT vs JNK1 KO MACS-sorted NG2^+^ OPCs (red). DAPI (blue) counterstains cell nuclei. (**F–I**) Quantification, through Neurolucida reconstruction, of soma areas (**F**), number of primary ramifications (**G**), total length (**H**) and complexity (**I**) of the ramifications of WT vs JNK1 KO MACS-sorted OPCs. (**J**) Sholl analysis of WT vs JNK1 KO MACS-sorted OPCs. Asterisks in (**I**) and (**J**): Two-way ANOVA (main effect of genotype). Scale bars: 50 μm in (**A**) and in (**D**). *WT* wild type, *NG2* neural/glial antigen 2, *Ki67* Ki67 antigen. *P < 0.05; **P < 0.01; ****P < 0.0001.

In culture, MACS-sorted JNK1 KO OPCs showed higher cell densities per field (Fig. 4A–D) and a twofold higher proliferation rate compared to WT cells, as revealed by colocalization with the proliferative marker Ki67 (Fig. 4A,B). Of note, while the proliferative fraction of WT cells decreased with increasing cell densities, the proliferative fraction of JNK1 KO OPCs remained constant, irrespective of the number of OPCs (Fig. 4C,D). As regards apoptosis, we found a threefold higher fraction of cCASP3^+^ JNK1 KO OPCs compared to WT cells and an apoptotic rate decreasing with increasing densities in both KO and WT cells (Suppl. Fig. 4A,B). These data suggest that, although increased in KO cells, apoptosis is similarly regulated in both mutant and WT cells, whereas in mutant OPCs proliferative regulatory mechanisms may be altered as a consequence of JNK1 loss.

Moreover, morphometric analyses on non-proliferative isolated OPCs showed that JNK1 KO OPCs display a reduced ramification complexity compared to WT cells (Fig. 4E,F–J) in the presence of similar soma area (Fig. 4F) and of a slightly higher number (about 12%) of primary ramifications (Fig. 4G). These results reveal that mutant OPCs show alterations independently of the presence of other cell types.

**Figure 5:**
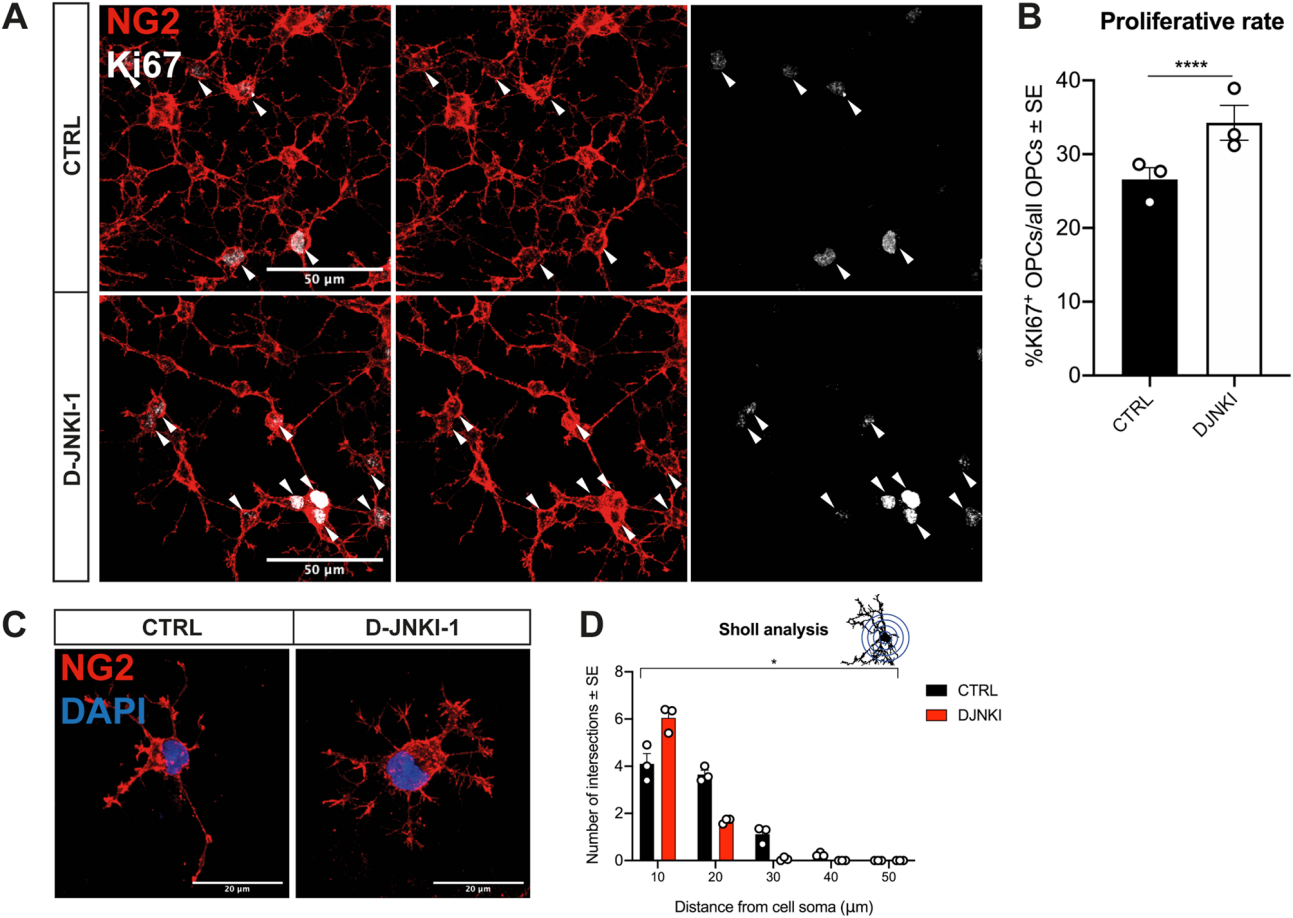
D-JNKI-1 treatment of rat OPCs mimics JNK1 KO in vitro. (**A**) Representative images and (**B**) quantification of proliferative rat NG2^+^ CTRL vs D-JNKI-1-treated OPCs (red). Ki67^+^ proliferating cells (white) are indicated by white arrows. (**C**) Representative images and (**D**) Sholl analysis of CTRL vs D-JNKI-1-treated OPCs (red). DAPI (blue) counterstains cell nuclei. Asterisks in (**D**) Two-way ANOVA (main effect of genotype). Scale bars: 50 μm in (**A**) and 20 μm in (**C**). *CTRL* control cells, *D-JNKI-1* JNK1 inhibitor-treated cells, *NG2* neural/glial antigen 2, *Ki67* Ki67 antigen. *P < 0.05; **P < 0.01; ****P < 0.0001.

To confirm these results in a distinct experimental model, we investigated the effects of JNK inhibition obtained with the D-JNKI-1 inhibitor^36^ on rat OPC cultures. D-JNKI-1 is a cell penetrating peptide that prevents, through a competitive mechanism, the binding of JNK to both the scaffold protein JNK-interacting protein-1 (JIP1) and its substrates^36–38^. Of note, D-JNKI-1 does not act exclusively on the binding of JNK1, but also on that of JNK2 and JNK3. Analysis of Ki67 expression revealed a higher proliferative rate in OPCs treated with D-JNKI-1 compared to controls (Fig. 5A,B). Moreover, morphometric analyses highlighted branching alterations resulting in a reduced ramification complexity (Fig. 5C), thus resembling those of MACS-sorted JNK1 KO OPCs, as indicated by Sholl analysis (Fig. 5D).

Altogether these data show that JNK1 KO-related OPC functional and morphological abnormalities occur also independently of other cell types affected by the mutations and suggest that JNK1 is implicated in the regulation of OPC proliferation and process architecture through a cell autonomous mechanism.

### JNK1 KO OLs do not show overt differentiation defects in vitro but display reduced territory occupancy

In order to study whether JNK1 KO myelin alterations in vivo could be explained by an altered ability of JNK1 KO OLs to differentiate, we cultured MACS-isolated OPCs derived from P0 WT or JNK1 KO in non-proliferative conditions and examined MBP expression as well as cell morphology.

**Figure 6:**
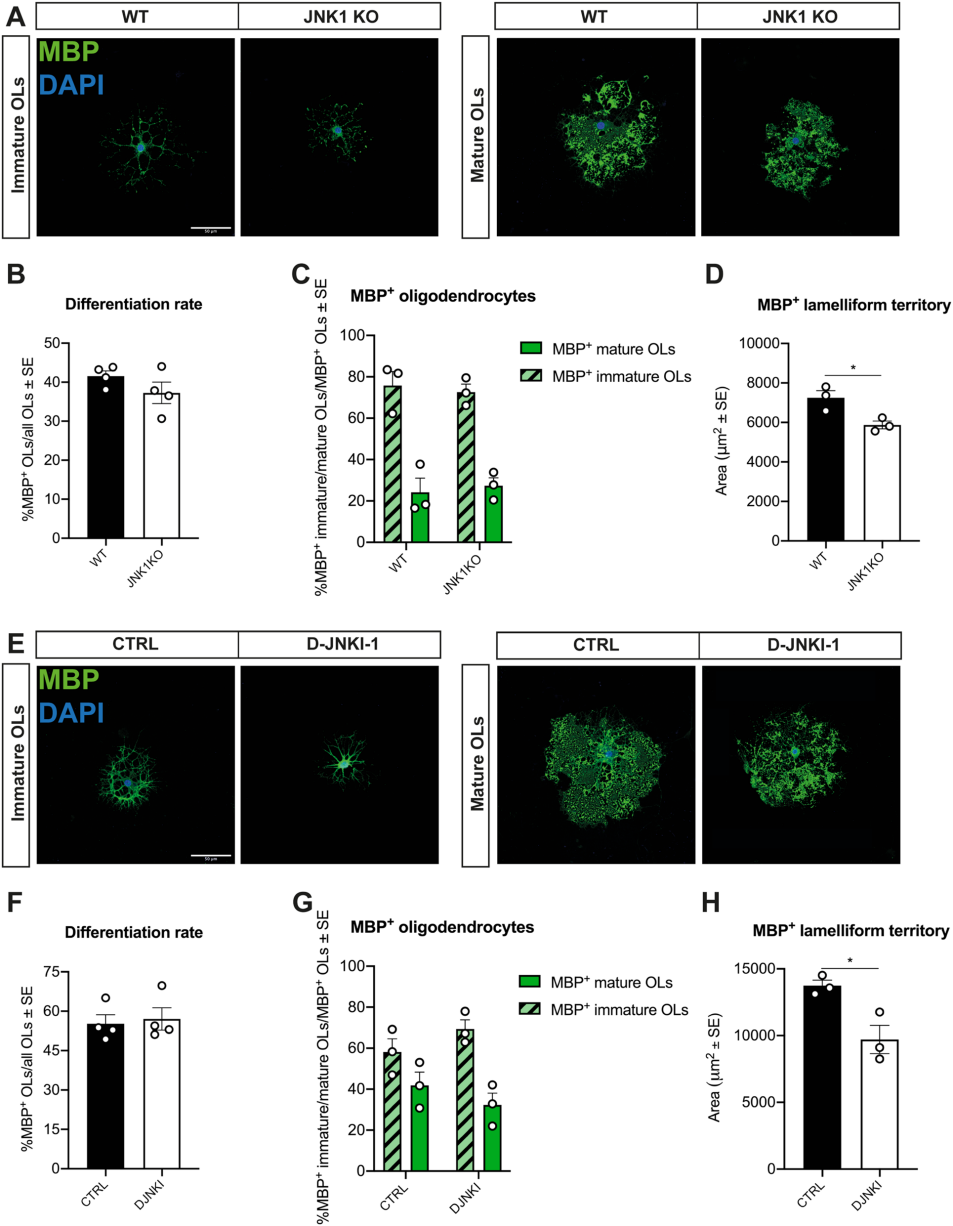
JNK1 KO and D-JNKI-1 treated OLs can differentiate and produce MBP*.* (**A**) Representative images and (**B**) quantification of differentiated MBP^+^ WT vs JNK1 KO OLs (green). DAPI (blue) counterstains cell nuclei. (**C**) Quantification of the percentage of immature and mature WT vs JNK1 KO OLs. (**D**) Quantification of the MBP^+^ lamelliform territory occupied by WT vs JNK1 KO OLs. (**E**) Representative images and (**F**) quantification of differentiated MBP^+^ CTRL vs D-JNKI-1 treated OLs (green). DAPI (blue) counterstains cell nuclei. (**G**) Quantification of the percentage of immature and mature CTRL vs D-JNKI-1 treated OLs. (**H**) Quantification of the MBP^+^ lamelliform territory occupied by CTRL vs D-JNKI-1 treated OLs. Scale bars: 50 μm in (A) and (E). *WT* wild type, *MBP* Myelin Basic Protein, *CTRL* control cells, *D-JNKI-1* JNK1 inhibitor-treated cells. *P < 0.05.

JNK1 KO and WT OLs in culture displayed equivalent capability to express MBP (Fig. 6A,B). Moreover, when we analyzed the frequency of immature vs mature MBP^+^ cells, as distinguished by process complexity and by MBP localization (see “Methods”) we found no differences in JNK1 KO vs WT cells (Fig. 6C).

We also investigated the effects of JNK inhibition obtained with D-JNKI-1^36^ on cultured rat OLs. Analysis of MBP^+^ OLs confirmed the results obtained for mutant OLs showing that D-JNKI-1 treated cells were able to differentiate, branch and form MBP^+^ lamellae to the same extent of control cells (Fig. 6E–G). However, in both experimental conditions, the cell territory occupied by MBP^+^ lamellae appeared reduced in JNK1 KO and D-JNKI-1 treated OLs (Fig. 6D,H).

Overall, these data show that the germinal ablation/inhibition of JNK1 does not affect the ability of OPCs to differentiate in MBP^+^ OLs.

## Discussion

The ERK/MAPK pathway is known to take part in the regulation of OPC architecture, proliferation and oligodendro-/myelino-genesis^11, 12^. Among MAPKs, JNK1 contribution to oligodendroglial biology has been only marginally investigated so far. In this study, we found that constitutive JNK1 ablation in KO mice is associated with decreased expression of myelin proteins and myelin/paranodal abnormalities in the cerebral cortex and CC of postnatal and adult mice. Such alterations are accompanied by a transient increase in OPC density and proliferative ability and by a persistent reduction in OPC ramifications complexity. These abnormal features are also present in JNK1 KO OPCs cultures and in WT OPCs cultures treated with D-JNKI-1, indicating that cell types distinct from oligodendroglial cells are not implicated in such alterations and suggesting a cell autonomous role of JNK1 in OPCs. On the other hand, JNK1 KO cultured OLs and D-JNKI-1 treated cells progress toward differentiation similarly to WT cells but displayed reduced lamellae-occupied territories, thus suggesting a cell-autonomous contribution of JNK1 in the observed cortical/CC hypomyelination.

### JNK1 and myelination

In the cerebral cortex of the mutant mice, we observed a lower expression of myelin proteins and longer CASPR^+^ paranodes, suggesting deficits in myelin structure and alterations in myelinating OLs/axon crosstalk. Defective myelin deposition and alterations in the paranode length are two recurrent features of hypo/dysmyelinating conditions linked to primary oligodendroglia pathology^30, 31^. In vitro experiments indicate that JNK1 KO does not impair MBP expression or affect major steps of OL differentiation. However, morphological maturation in differentiating OLs appeared affected in mutant and treated cells, as lamellae-occupied territories were reduced. Based on evidence that OL morphological or cytoskeletal alterations are often associated with reduced myelination^9, 39^, it is conceivable that reduced membrane extension in mutant OLs may contribute to the hypomyelinated phenotype found in vivo. Although we did not observe overt degeneration in JNK1 KO axons at the examined ages and territories, former electron microscopy investigations revealed some extent of axonal degeneration in JNK1 KO mice^17^, and showed that JNK1 takes part in microtubule maintenance and integrity, since earlier ages^16, 17^. Microtubule dynamics both in neurons and oligodendrocytes play a fundamental role in OLs/neuron crosstalk, whose integrity is crucial for a correct myelination^40–43^. On these bases, we cannot exclude subtle microtubule-related alterations in axons, myelin sheath formation and/or OLs/axon crosstalk could all take part in the hypomyelination phenotype observed in vivo in JNK1 KO. Further investigations are needed to clarify this issue.

### JNK1 role in OPC proliferation and apoptosis

We also found that JNK1 KO OPCs display a higher proliferative rate associated with increased density at postnatal developmental stages, with no changes in their distribution through cortical layers. This feature suggests that JNK1 operates as a negative regulator of cell proliferation in OPCs. According to in vitro experiments JNK1 appears to act in a cell autonomous fashion in cell division regulation in OPCs. However, it cannot be excluded that JNK1 KO OPCs could have been primed to an altered regulation of proliferation by environmental signals received at embryonic ages in vivo, so to determine their increased division rate also in purified culture conditions.

Notably, our observations apparently clash with the results of former studies showing JNK pathway (although without isoform specifications) as necessary for OPC proliferation upon incubation with the conditioned medium of neuroblastoma cells^44^. However, on the other hand, JNK1 specific inhibition was shown to increase endothelial cell division in controlled conditions^45, 46^ or to have no effect in a carcinoma cell line^47^. Moreover, in cancer development, JNK1 seems to play a dual role in promoting/inhibiting cell proliferation^48^. Thus, literature data indicate a cell/context dependent role for JNK1 in the modulation of proliferative events.

OPC proliferation is finely tuned by two main mechanisms. One first mechanism appears to operate through an intracellular timer driven by the mitogen PDGF (Platelet-Derived Growth Factor), that determines when individual OPCs should stop dividing to proceed toward differentiation^49–51^. One other mechanism implies OPC-to-OPC contact-mediated inhibition of cell proliferation through, for instance, Netrin-1 (NT-1) and its receptor Deleted in Colorectal Cancer (DCC) signaling^6, 7^. Of note, other sources of these contact-mediated inhibitors are unclear, although neurons have been shown to produce NT-1^7, 52^. Former studies have implicated JNK1 activity as a positive regulator of cell cycle progression and a mediator of PDGF actions in OPCs^53^. On the other hand, JNK1 was also reported to mediate NT-1/DCC signaling in neurons, suggesting that similar mechanisms could act also in oligodendroglia and, therefore, that JNK1 ablation could alter contact-mediated OPC proliferation inhibition^54^. In vitro data appear to support this latter hypothesis, as they show that, at difference with WT cells, JNK1 KO OPC proliferative rate is maintained high also in conditions of elevated cell density.

Our data further showed that JNK1 KO OPCs proliferation and density in vivo are increased only during developmental stages. Although OPC amplification, self-maintenance and maturation at adult stage are supposed to recapitulate the corresponding developmental processes^55^, to what extent the very same molecular mechanisms subserve these events in the postnatal *vs* adult CNS is unclear. Age-dependent differences in gene expression and function occur in OPCs. In particular, early OPCs are more proliferative, characterized by a shorter cell cycle and more susceptible to JNK-dependent death^24, 56–58^. Whether and how JNK1 is involved in postnatal *vs* adult OPC distinct properties remains elusive. We can also speculate that supernumerary JNK1 KO OPCs may be simply eliminated in parallel with the progression of myelination, thereby adjusting the number of OLs to that of the axons (and to limiting amounts of trophic factors provided by axons)^59^, as normally occurs in WT brains^60, 61^.

JNK1 signaling has also been reported to participate in cell death which could impact on proliferation rates and cell densities. JNK pathway was shown to promote apoptosis in OPCs/OLs under stress conditions^62–64^. However, if JNK1 isoform is implicated in physiological cell death is unknown. In in vivo analyses we did not find evidence of an altered apoptosis rate in JNK1 KO OPCs. Conversely, in MACS-sorted JNK1 KO OPCs cultures, we found an increased fraction of apoptotic cells. Such a fraction, similar to what occurs for WT cells, appeared to decline with increasing cell densities, in agreement with an increased production of survival signals at sites with high cellularity. These data overall suggest that the mechanisms underlying the physiological regulation of apoptosis are maintained in mutant cells, and increased apoptosis may simply reflect the increased number of JNK1 KO OPCs. This may imply that, in OPCs, JNK isoforms other than JNK1 regulate this aspect, or can compensate for JNK1 ablation in the physiological regulation of apoptosis.

### JNK1 role in OPC architecture

Our analyses also provided evidence of a transient alteration of OPC territory occupancy. Voronoi polygons and cell territory analyses (Fig. 3F–I,K,L) show that, at least during development, OPC territory in JNK1 KO is significantly reduced. Although at adult stages this gross OPC alteration seems to be restored, adult JNK1 KO OPCs displayed a reduction in ramification length and branching complexity (Fig. 3J–O). These defects were also recapitulated in cell culture analyses (Figs. 4E–J, 5C–D), confirming the cell autonomous role of JNK1. These findings might also reflect the persistence of less complex immature phenotypes associated with the increased proliferative activity of the mutant cells. However, the maintenance of morphological alterations at adult ages, when mutant cell proliferation has declined back to WT levels, supports an involvement of JNK1 in OPC cytoskeletal dynamics independent of proliferative events, as previously found in neurons^17, 65^. In keeping with this possibility, one potential JNK1 effector candidate in the regulation of OPC cytoskeleton is the microtubule-associated protein 1B (MAP1B), expressed both in neurons and oligodendrocytes^66^, that regulates microtubule elongation and dynamics. MAP1B is activated by kinases including JNK through phosphorylation^67^, and in neurons is known to support axon outgrowth. Notably, among the JNK isoforms, JNK1 appears to be particularly involved in the process of axonal elongation^68^. In oligodendroglia, MAP1B is expressed in OPCs progressing toward the preoligodendrocyte stages^66, 69, 70^—a transition that involves profound morphological changes—suggesting that its deregulated activation in the absence of JNK1 could participate in the altered branching of mutant OPCs. Another possible target of JNK1 in the regulation of OPC cytoskeleton is mTOR. Both molecules act in parallel or via cross-regulation in many pathological contexts, where JNK seems to positively regulate mTOR activity^71^.

### JNK isoform 1 appears to play a predominant role in oligodendroglia

In further support of JNK1 functions in oligodendrocytes, we found that mutant cell proliferative and morphological alterations were recapitulated in WT OPCs treated with D-JNKI-1^36^. This inhibitor is able to block JIP-JNK interaction, thus preventing the phosphorylation of c-Jun, the main downstream target of all JNK isoforms, and of the other JBD targets^38, 72^. Thus, this treatment might have revealed a much broader impact on the cells. However, D-JNKI-1 administration well recapitulated the proliferative and morphological phenotype of JNK1 KO OPCs, suggesting that JNK1, among the three JNK isoforms, has a predominant role in the regulation of OPC proliferation, branching and membrane extension. This hypothesis is also supported by qRT-PCR data of MACS-sorted OPCs (Suppl. Fig. 4C–E), revealing the absence of any compensatory upregulation or dysregulated expression the other two JNK isoforms.

In conclusion, our study shows that JNK1 ablation results in persistent myelin abnormalities in vivo and that JNK1 participates in a cell-autonomous manner in the regulation of OPC proliferation, branching architecture and membrane extension at mature stages, unveiling a novel player in the complex regulatory network of OPC biology. Further investigations are needed to disentangle the potential contribution of axonal vs oligodendroglial alterations in the hypomyelination phenotype in vivo. Finally, it is also interesting to note that most of the alterations that we reported in mutant oligodendroglia (i.e. contact-mediated regulation of OPC proliferation, OPC branching architecture and paranodal organization) are regulated by NT-1 signaling^7, 73–75^. Further studies are needed to clarify whether NT-1 acts via JNK1 in oligodendroglia, as formerly shown in neurons^76, 77^.

## Methods

### Experimental animals

For histological analyses and Magnetic-Activated Cell Sorting (MACS) we employed JNK1 KO^78^ and age-matched wild-type (WT) mice as controls. Perfusions of juvenile and adult mice were carried out under deep general anaesthesia obtained by intraperitoneal administration of ketamine (100 mg/kg; Ketavet; Bayern; Leverkusen, Germany) supplemented by xylazine (5 mg/kg; Rompun; Bayer). For OPCs cultures, postnatal (P0-P1) mice and rats were anesthetized on melting ice. Groups of 4–5 mice were housed in transparent polycarbonate cages (Tecnoplast, Buggirate, Italy) provided with sawdust bedding, boxes/tunnels hideout as environmental enrichment and striped paper as nesting material. Food and water were provided ad libitum; environmental conditions were 12 h/12 h light/dark cycle, room temperature 21 °C ± 1 °C and room humidity 55% ± 5%.

The experimental plan was designed according to the guidelines of the NIH, the European Communities Council (2010/63/EU) and the Italian Law for Care and Use of Experimental Animals (DL26/2014). It was also approved by the Italian Ministry of Health (Authorization 1112/2016 prot E669C.20) and the Bioethical Committee of the University of Turin. The study was conducted according to the ARRIVE guidelines.

### Histological procedures

Animals were anaesthetized and transcardially perfused with 4% paraformaldehyde (PFA) in 0.1 M phosphate buffer (PB). Brains were post-fixed for 1 or 5 h (for immunofluorescence or Nissl/Gallyas staining, respectively), cryoprotected, and processed. Brains were cut in sagittal sections, for Gallyas stain of myelin using silver nitrate^79, 80^. Otherwise, brains were cut in 40 µm thick coronal sections and then treated in order to detect the expression of the following antigens: NG2 (1:200, Millipore, Billerica, MS, USA); PDGFRα (APA-5 clone, 1:300, BD Biosciences, San Jose, CA, USA); MBP (Smi-99 clone, 1:1000 Sternberger); SMI31 (1:500, SMI-31R Sternberger); CASPR (1:1000, Abcam); cCASPase-3 (1:200, Cell Signaling); Ki67 (1:750, Invitrogen). Incubation with primary antibodies was made overnight at 4 °C in PBS with 2% Triton-X 100. The sections were then exposed for 2 h at room temperature (RT) to secondary Cy3−/ Cy2− (Jackson ImmunoResearch Laboratories, West Grove, PA), Alexafluor 647- (Molecular Probes Inc, Eugene, Oregon) conjugated antibodies. 4,6-diamidino-2-phenylindole (DAPI, Fluka, Milan, Italy) was used to counterstain cell nuclei. Sections were mounted on microscope slides with Tris-glycerol supplemented with 10% Mowiol (Calbiochem, LaJolla, CA). TUNEL assay was performed using the TMR red In Situ Cell Death Detection Kit (Roche, Basel, Switzerland) according to the manufacturer’s protocol.

### Cell cultures

After tissue dissociation with a papain + DNAseI solution (papain 1.5 mg/ml, l-cysteine 360 µg/ml, DNAseI 1000U/ml in MEM; all from Sigma-Aldrich, Saint Louis, MS, USA), mouse OPCs were enriched by positive selection using an anti-PDGFRα antibody conjugated to magnetic beads, according to the instructions of the manufacturer (Miltenyi Biotech GmbH, Bergisch Gladbach, DE). MACSorted OPCs were plated onto poly-d-lysine (1 µg/ml, Sigma-Aldrich, Saint Louis, MS, USA) coated glass coverslips in a proliferative medium including Neurobasal, 1X B27 (Invitrogen, Milan, Italy), 2 mM l-glutamine (Sigma-Aldrich, Saint Louis, MS, USA), 10 ng/ml PDGF-BB and 10 ng/ml human bFGF (Miltenyi Biotech GmbH, Bergisch Gladbach, DE), or processed for quantitative RT-PCR analysis. Purity of the MACS-selected OPCs was verified by immunocytochemistry (more than 95% of the cells were NG2-positive (^+^) at 6 h post-plating). Cells were cultured 3DIV in proliferative medium (described above) and fixed. Alternatively, they were maintained 1DIV in proliferative medium and 6DIV in non-proliferative medium—Neurobasal, 1X B27 (Invitrogen, Milan, Italy), 2 mM l-glutamine (Sigma-Aldrich, Saint Louis, MS, USA)—to allow differentiation, and subsequently fixed.

To test the effect of D-JNKI-1 treatment, primary rat OPCs were isolated by the shaking method from mixed glial cultures obtained from P0-1 Sprague–Dawley rat cortex, as described in^5^. OPCs were plated onto poly-D-lysine (1 µg/ml, Sigma-Aldrich) -coated 12-mm glass coverslips for immunocytochemistry (5 × 10^4^ cells/coverslip) in the proliferative medium (see above). After 1 day in vitro (DIV), the cell permeable JNK-inhibitor D-JNKI-1 (2 µM)^36^ was added to the medium until fixation (after 3DIV or 7DIV in proliferative or non-proliferative conditions, respectively).

After 3 or 7 DIV, OPCs/OLs (both from mice and rats) were then fixed for 20 min at RT with 4% PFA in 0.1 M PB and labelled with anti-NG2 (1:400, Millipore, Billerica, MS, USA), -Ki67 (1:1000, Novocastra), -cCASPase-3 (1:400, Cell Signaling) and -MBP (Smi-99 clone, 1:1000 Sternberger) antibodies overnight at 4 °C in PBS with 0.25% Triton-X. Then, coverslips were incubated with Cy3−/Cy2− (Jackson ImmunoResearch Laboratories, West Grove, PA) and Alexafluor647- conjugated secondary antibody (Molecular Probes, Eugene, Oregon) for 1-h RT. After a 5-min incubation with DAPI (1:1000, Fluka, Saint Louis, USA), coverslips were mounted with Tris-glycerol supplemented with 10% Mowiol (Calbiochem, LaJolla, CA).

### In vivo cell counting, 3D reconstructions, densitometric and morphological analyses

Histological specimens were examined using a Leica TCS SP5 (Leica Microsystems, Wetzlar, Germany) confocal microscope. Quantitative evaluations (i.e. PDGFRα^+^ cell density, density and fraction of cell duplets, NG2^+^/Ki67^+^ cell density, cCASP3^+^/NG2^+^ cell density, 3D-reconstruction of the corpus callosum) were performed by means of the Neurolucida system (MicroBrightfield, Colchester, VT).

The extent of MBP/SMI31 or CASPR staining was quantified with ImageJ (Research Service Branch, National Institutes of Health, Bethesda, MD; available at http://rsb.info.nih.gov/ij/) as percentage of positive pixels over an area of 0.15 mm^2^ in confocal image stacks comprising 16 optical slices 0.99 µm thick (for MBP/SMI31) or over an area of 0.015 mm^2^ in confocal image stacks comprising 5 optical slices 0.99 µm thick (for CASPR). Confocal images (1024 × 1024 pixels) of MBP/SMI31 or CASPR immunostaining were all acquired with a speed of 200 Hz with the same settings (i.e. pinhole size: MBP, 67.9 µm; SMI31, 67.9 µm; CASPR, 67.9 µm; laser power: MBP, 80%; SMI31, 28%; CASPR, 10%; gain: MBP, 484.0 V; SMI31, 570.0 V; CASPR, 570.0; offset: MBP, − 2%; SMI31, − 1.4%; CASPR, − 3%) and analyzed after ImageJ default auto-thresholding (i.e. IJ_IsoData). Voronoi analysis of the cell distribution was performed with ImageJ while cell territory and soma area were analyzed with Imaris (Bitplane) software (only cells whose entire extension was completely included in the confocal stack were considered). The number of inspected cells ranged from 46 to 70 cells per individual, with a total of ~ 300/350 cells per genotype. Primary ramifications, ramification length and complexity of branching analyses were performed with the Neurolucida system (MicroBrightfield, Colchester, VT). The analysis of the complexity of branching was performed assigning progressive numbers (i.e. orders) to branches extending directly from the cell soma (order 1) and then to all processes centrifugally emerging from subsequent branches (order > 1), to describe the hierarchy of the branching scheme. Each tree (i.e. each primary ramification (order 1) associated with its branching scheme) was analyzed individually. Plotted values (Figs. 3O,4I) represent the mean of all analyzed trees. OPCs juxtaposed with symmetrical cell somata and decondensed grainy DNA were recognized as duplets of cells that exited cytokinesis after cell division^5, 81, 82^. As such, OPC duplets were counted in tissue slices to measure OPC proliferative activity^32^. OPC proliferation was also evaluated by counting NG2^+^/Ki67^+^ double positive cells. Adobe Illustrator 6.0 (Adobe Systems, San Jose, CA) was used to assemble the final plates. In all the analyses the experimenter was blind to the genotype of the samples.

### In vitro cell counting and morphological analyses

Expression of Ki67/cCASP3 in cultured OPCs and of MBP in cultured OLs was investigated live in five to eight quadrants localized in central and peripheral areas of each coverslip—as described in^83^—with the Neurolucida software. Results for each quadrant were expressed as a percentage of marker-positive cells over the number of OPCs and averaged across different coverslips. For reconstructing OPC arborizations, 20–30 non-proliferative (Ki67- negative) OPCs/coverslip isolated from other cells were randomly selected and traced live with the Neurolucida software, with a total of ~ 60–70 inspected cells per condition. Cultured MBP^+^ OLs were categorized in immature/mature OLs depending on the localization of MBP^+^ staining (restricted to ramifications for immature cells or further expanded to lamellae-like membranes for mature cells) and on the complexity of their processes (poorly branched for immature cells and complexly branched for mature cells, as described in^84^). The surface occupancy of MBP^+^ lamelliform OLs was analyzed with ImageJ. The number of inspected cells ranged from 15 to 25 cells per coverslip, with a total of ~ 80/100 cells per condition. In all cell counting and morphological analyses the experimenter was blind to the genotype or treatment of the cells.

### Quantitative RT-PCR

Total RNA from MACS-sorted OPCs was extracted with the Direct-zol RNA Miniprep kit (Zymo Research, Irevine, USA), and reverse transcribed to cDNA with the High-Capacity cDNA Archive kit (Applied Biosystems, Thermofisher, Waltham, USA). Quantitative Real Time RT-PCR was performed as described in^85^, either with pre-developed Taqman assays (Applied Biosystems, Thermofisher, Waltham, USA) or by combining the RealTime Ready Universal Probe Library (UPL, Roche Diagnostics, Monza, Italy) with the primers indicated in Suppl. Table 1. A relative quantification approach was used, according to the 2^-ddCT^ method^86^. β-actin was used to normalize expression levels.

### Tissue dissection, lysates and western blotting

CC and cortices from P7, P15 and P30 WT and JNK1KO mice, after brain sectioning with Leica vibratome, were obtained by dissection. Tissue lysates were obtained adding RIPA buffer (1% NP40, 150 mM NaCl, 50 mM TRIS HCl pH 8, 5 mM EDTA, 0.01% SDS, 0.005% Sodium deoxycholate, Roche protease inhibitors, PMSF) for 10 min at 4 °C. Samples were homogenized on ice with a pellet pestle (Sigma-Aldrich, Saint Louis, MS, USA) and centrifuged at 1300 rpm at 4 °C. For immunoblots, equal amounts of proteins were resolved by SDS–PAGE and blotted to nitrocellulose membranes, which were then probed with anti-MBP (1:1000, Millipore, Billerica, MS, USA—MW: 18–21 kDa), -CNPase (1:500, Sigma-Aldrich, Saint Louis, MS, USA—MW: 47 kDa), -MOG (1:1000, Proteintech, Manchester, UK—MW: 25 kDa) and -SMI31 (1:1000, SMI-31R Sternberger—MW: 160–200 kDa) antibodies. The membranes were subsequently incubated with the secondary antibodies and developed using the Luminata Forte HRP substrate (Millipore, Billerica, MS, USA). Signals are normalized using anti-β-Tubulin (1:5000, Sigma-Aldrich, Saint Louis, MS, USA—MW: 50 kDa) and anti-Vinculin (1:2000, Sigma-Aldrich, Saint Louis, MS, USA—MW: ~ 120 kDa) antibodies. Blots were imaged on a ChemiDoc (Bio-Rad) and analyzed using Image Lab software.

### Statistical analyses

In all histological quantifications, at least three animals and three sections per animal were analyzed for each time point and experimental condition. Western blotting analyses were performed with three animals for each time point and experimental condition, with at least three technical replicates. For in vitro experiments, at least three experiments were performed, each with at least two technical replicates per condition. Statistical analyses were carried out with GraphPad Prism 7 (GraphPad software, Inc). The Shapiro–Wilk test was first applied to test for a normal distribution of the data. When normally distributed, unpaired Student’s t test (to compare two groups) and Two-way ANOVA test (for multiple group comparisons) followed by Sidak’s post hoc analysis were used. Statistics also included Chi-square test (to compare frequencies) and linear regression analysis (to analyze in vitro OPC proliferation and apoptosis in relation to cell density). In all instances, P < 0.05 was considered as statistically significant. Histograms represent mean ± standard error (SE). Statistical differences were indicated with *P < 0.05, **P < 0.01, ***P < 0.001, ****P < 0.0001. The list of the applied tests, F values and values for n (animals for in vivo analyses, experiments for in vitro analyses), results of post hoc analyses are included in Supplementary Table 2. Supporting data are available within the article or can be provided upon request.

## Supplementary Information

Supplementary Information.
