# Supplementary material for: c-Jun N-terminal kinase 1 (JNK1) modulates oligodendrocyte progenitor cell architecture, proliferation and myelination
Source: Sci Rep. 2021 Mar 31;11:7264. doi: 10.1038/s41598-021-86673-6 (PMC8012703; doi:10.1038/s41598-021-86673-6)
Supplement: Supplementary file 1 — Supplementary Information. [file 41598_2021_86673_MOESM1_ESM.pdf]

c-Jun N-terminal Kinase 1 (JNK1) modulates oligodendrocyte progenitor cell architecture, proliferation and myelination

Martina Lorenzati[1,2], Enrica Boda[1,2], Roberta Parolisi[1,2], Martino Bonato[2], Tiziana Borsello[3,4], Thomas Herdegen[5], Annalisa Buffo[1,2,*] and Alessandro Vercelli[1,2,*]

Suppl. Figure 1

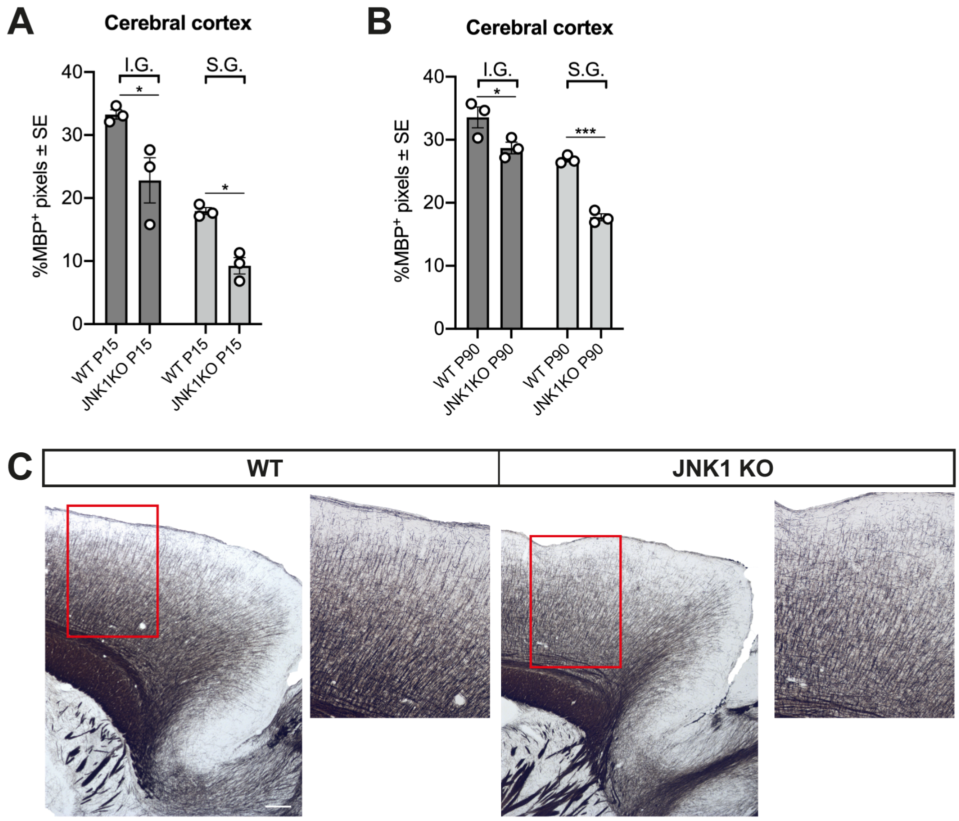

Supplementary figure 1.
(A-B) Quantification of the percentage of MBP+ pixels in P15 **(A)** and P90 **(B)** WT vs JNK1 KO mice infragranular (I.G) and supragranular (S.G.) layers. **(C)** Gallyas staining of sagittal P90 WT and JNK1 KO sections, highlighting myelinated cortical axons. Scale bars: 250µm in **(C)**. Abbreviations: WT, wild type; P, postnatal day; I.G., infragranular layers; S.G., supragranular layers; MBP, Myelin Basic Protein. *, P<0.05; ***, P<0.001.

Suppl. Figure 2

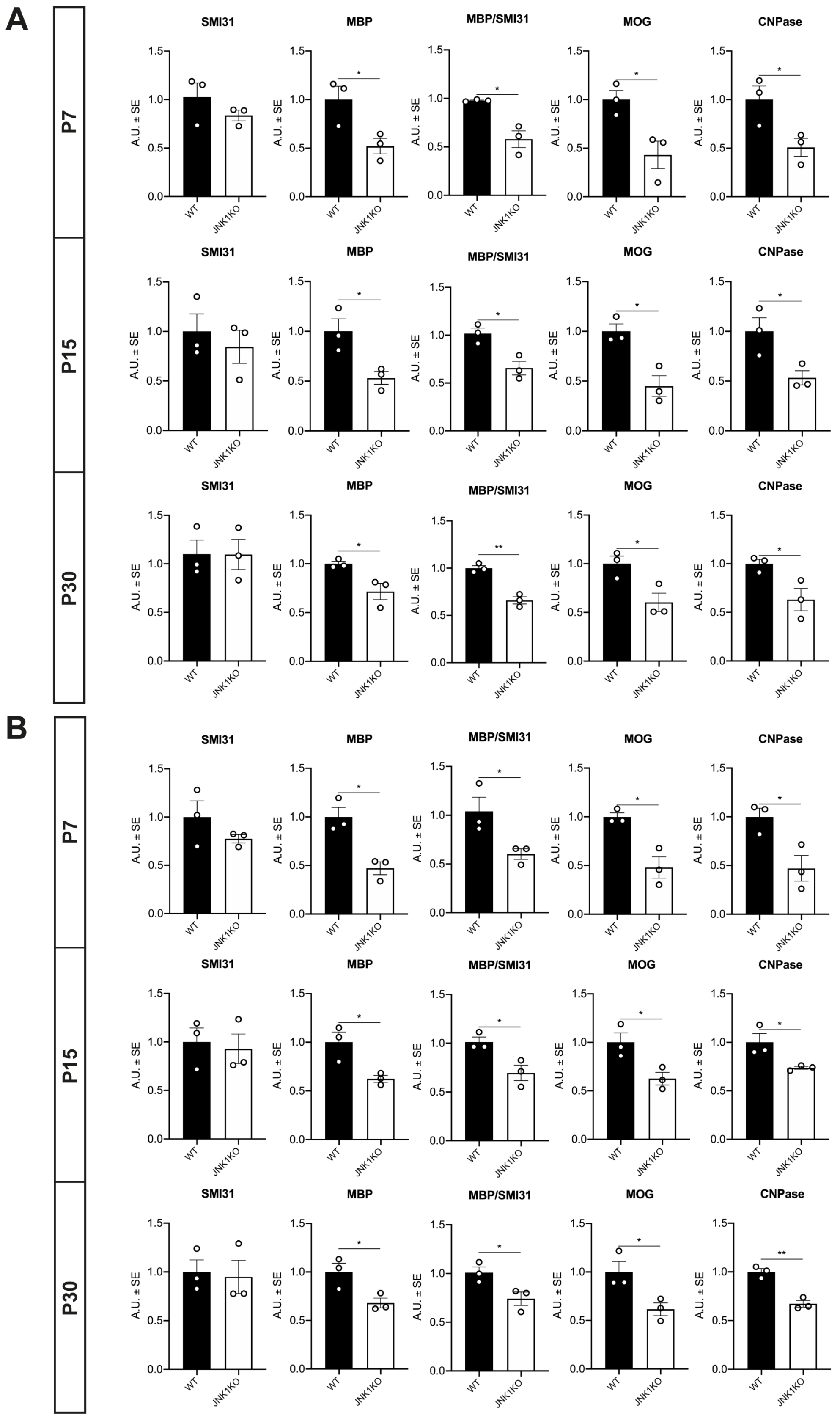

Supplementary figure 2.

(A-B) Quantification of the amount of SMI31, MBP, MOG and CNPase in P7, P15 and P30 WT and JNK1 KO cortices **(A)** and corpora callosa **(B)**. Ratio between MBP and SMI31 is shown. Abbreviations: WT, wild type; P, postnatal day; A.U., Arbitrary Units; SMI31, neurofilaments; MBP, Myelin Basic Protein; MOG, Myelin Oligodendrocyte Glycoprotein; CNPase, 2'-3'-Cyclic-nucleotide 3'-phodphodiesterase. *, $P<0.05$; ** $P<0.01$.

Suppl. Figure 3

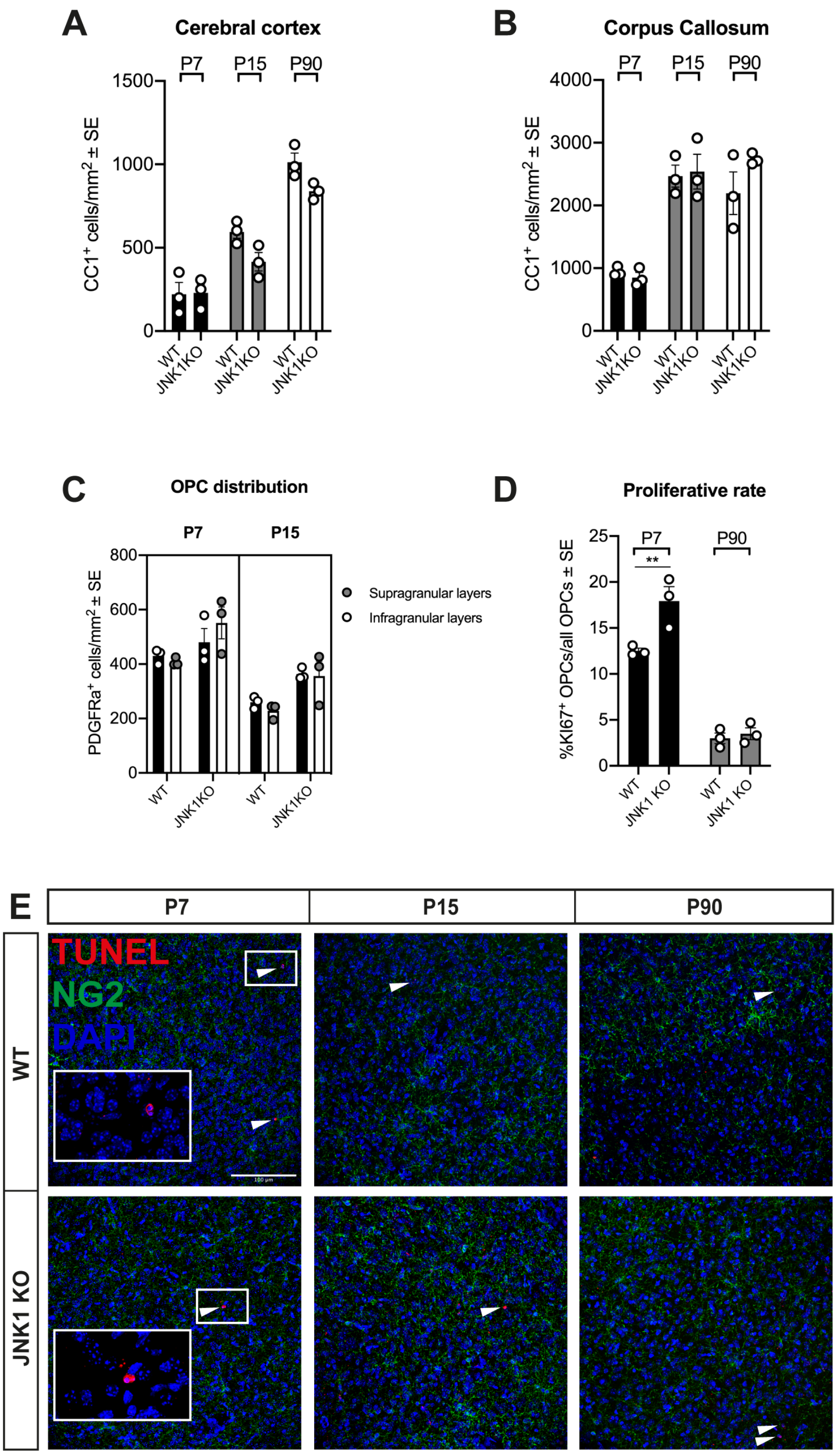

Supplementary figure 3.
(A) Quantification of the density of CC1+ myelinating oligodendrocytes of P7, P15 and P90 WT and JNK1 KO cortices and **(B)** corpora callosa. **(C)** Quantification of the density of PDGFRα+ OPCs throughout cortical supragranular and infragranular layers of P7 and P15 WT vs JNK1 KO mice. **(D)** Quantification of the Ki67+ proliferative fraction of NG2+ OPCs of P7 and P90 WT and JNK1 KO cortices. **(E)** Tunel assay on P7, P15 and P90 WT and JNK1 KO cortices. Scale bars: 100µm in (E). Abbreviations: WT, wild type; P, postnatal day; PDGFRα, platelet-derived growth factor receptor A. **, P<0.01.

Suppl. Figure 4

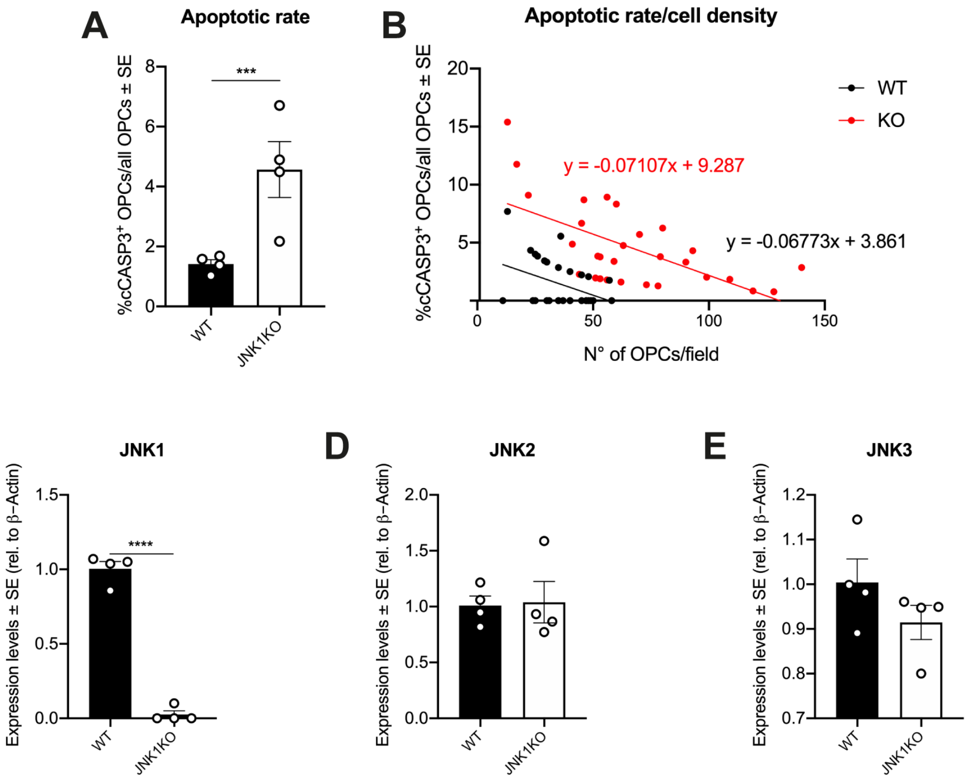

Supplementary figure 4.
(A) Quantification of the apoptotic rate in WT vs JNK1 KO MACS-sorted cultured OPCs. In **(B)** the apoptotic fraction (cCASP3+ OPCs over all OPCs) is plotted as a function of the number of OPCs in each analyzed field. **(C-E)** Quantification through rt-PCR of the expression levels of JNK1 **(C)**, JNK2 **(D)** and JNK3 **(E)** in MACS-sorted WT and JNK1 KO OPCs. Abbreviations: WT, wild type; P, postnatal day; cCASP3, cleaved caspase 3. ***, P<0.001; ****, P<0.0001.

Suppl. Figure 5

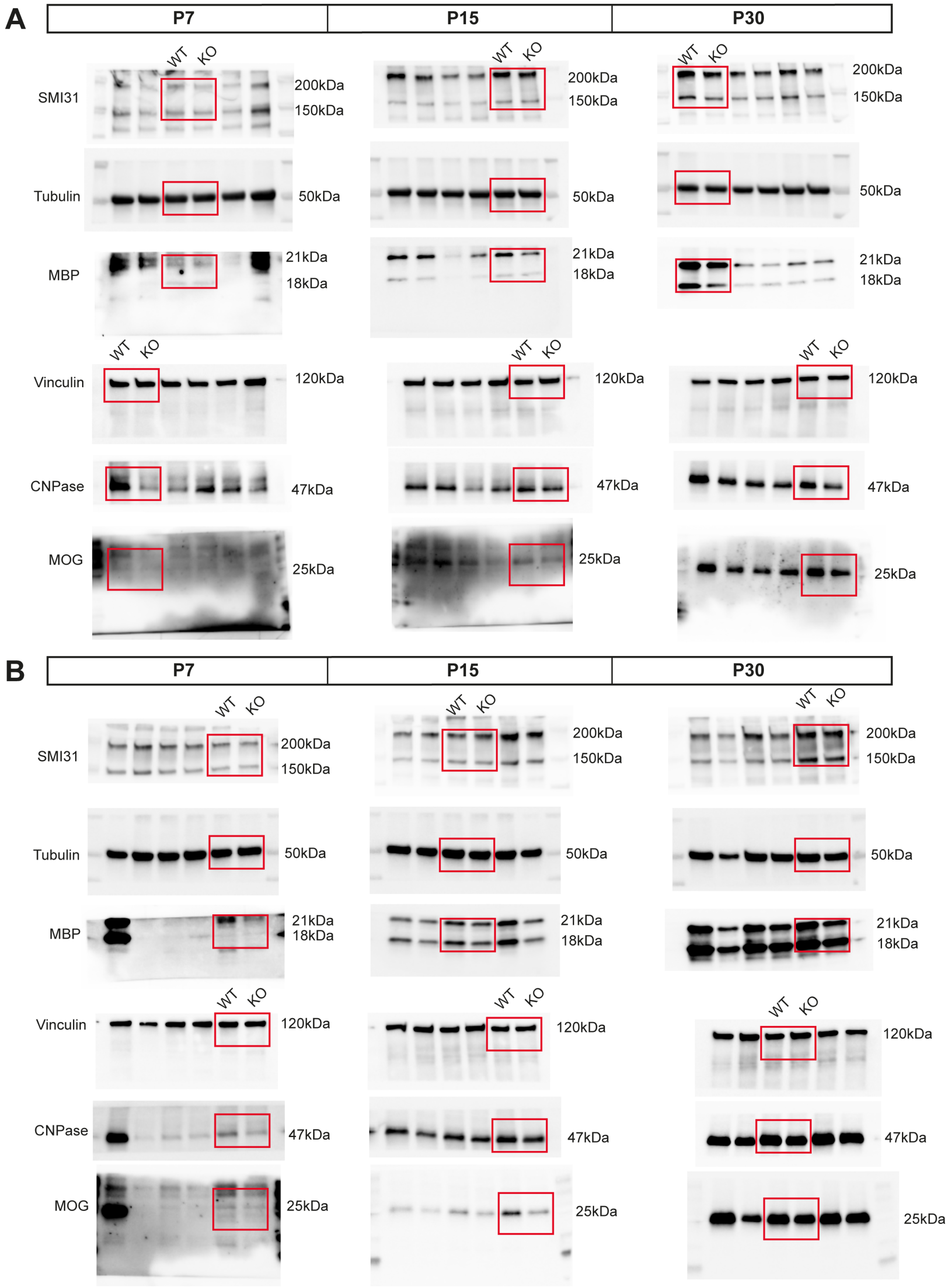

Supplementary figure 5.

(A-B) Full-length blots presented in Figure 2. **(A)** Full-length blots of P7, P15 and P30 WT and JNK1 KO (KO) cortices (presented in Figure 2E) and **(B)** corpora callosa (presented in Figure 2F). Abbreviations: WT, wild type; P, postnatal day; SMI31, neurofilaments; MBP, Myelin Basic Protein; MOG, Myelin Oligodendrocyte Glycoprotein; CNPase, 2’-3’-Cyclic-nucleotide 3’-phodphodiesterase.

Supplementary Table 1.

Gene	*Taqman assay (Applied Biosystems)*	*Primers+UPL probe (Roche Diagnostics)*
**β-actin (β-Act)**	Mm00607939_s1	
**c-Jun N-terminal Kinase 1 (JNK1)**		FW: aactgttccccgatgtgct RV: acaaatctcttgcctgactgg Probe #33
**c-Jun N-terminal Kinase 1 (JNK2)**		FW: tgactccctatgtggtaactcg RV: cacccgacagaccagatgt Probe #50
**c-Jun N-terminal Kinase 1 (JNK2)**		FW: tacgacccggctgaagtg RV: cattcttcgatggtgtgctc Probe #42

Supplementary Table 2. Statistics

Figure	Applied Test	n	P value	Statistics	Post hoc analyses	Post hoc results
**Fig. 1C**	Two-way Anova (2-tailed)	WT P7=3<br>JNK1 KO P7=3<br>WT P15=3<br>JNK1 KO P15=3<br>WT P90=3<br>JNK1 KO P90=3	**Genotype**<br>P<0.0001<br>**Age**<br>P<0.0001<br>**Interaction**<br>n.s.	**Genotype**<br>F(1,12)=49,68<br>**Age**<br>F(1,12)=56.10<br>**Interaction**<br>F(2,12)=1.199	Sidak's Multiple Comparison Test	WT P7 vs JNK1 KO P7 = P<0.05<br><br>WT P15 vs JNK1 KO P15 = P<0.001<br><br>WT P90 vs JNK1 KO P90 = P<0.01
**Fig. 1D**	Two-way Anova (2-tailed)	WT P7=3<br>JNK1 KO P7=3<br>WT P15=3<br>JNK1 KO P15=3<br>WT P90=3<br>JNK1 KO P90=3	**Genotype**<br>P<0.0001<br>**Age**<br>P<0.0001<br>**Interaction**<br>n.s.	**Genotype**<br>F(1,12)=33.02<br>**Age**<br>F(2.12)=23.37<br>**Interaction**<br>F(2.12)=0.3956.	Sidak's Multiple Comparison Test	WT P7 vs JNK1 KO P7 = P<0.05<br><br>WT P15 vs JNK1 KO P15 = P<0.01<br><br>WT P90 vs JNK1 KO P90 = P<0.05
**Fig. 1F**	Unpaired t test (2-tailed)	WT P90 =3<br>JNK1 KO P90 =3	P=0.0056	t(4)=5.416		
**Fig. 1G**	Unpaired t test (2-tailed)	WT P90 =3<br>JNK1 KO P90 =3	P=0.0329	t(4)=3.199		
**Fig. 2B**	Two-way Anova (2-tailed)	WT P7=3<br>JNK1 KO P7=3<br>WT P15=3<br>JNK1 KO P15=3<br>WT P90=3<br>JNK1 KO P90=3	**Genotype**<br>P<0.0001<br>**Age**<br>P<0.0001<br>**Interaction**<br>n.s.	**Genotype**<br>F(1,12)=42.77<br>**Age**<br>F(2.12)=51.80<br>**Interaction**<br>F(2.12)=0.01320	Sidak's Multiple Comparison Test	WT P7 vs JNK1 KO P7 = P<0.05<br><br>WT P15 vs JNK1 KO P15 = P<0.01<br><br>WT P90 vs JNK1 KO P90 = P<0.01
**Fig. 2C**	Two-way Anova (2-tailed)	WT P7=3<br>JNK1 KO P7=3<br>WT P15=3<br>JNK1 KO P15=3<br>WT P90=3<br>JNK1 KO P90=3	**Genotype**<br>n.s.<br>**Age**<br>P<0.0001<br>**Interaction**<br>n.s.	**Genotype**<br>F(1,12)=4.746<br>**Age**<br>F(2.12)=63.25<br>**Interaction**<br>F(2.12)=0.3420	Sidak's Multiple Comparison Test	WT P7 vs JNK1 KO P7 = n.s.<br><br>WT P15 vs JNK1 KO P15 = n.s.<br><br>WT P90 vs JNK1 KO P90 = n.s.
**Fig. 2D**	Two-way Anova (2-tailed)	WT P7=3<br>JNK1 KO P7=3<br>WT P15=3<br>JNK1 KO P15=3<br>WT P90=3<br>JNK1 KO P90=3	**Genotype**<br>P<0.0001<br>**Age**<br>P<0.0001<br>**Interaction**<br>n.s.	**Genotype**<br>F(1,12)=54.40<br>**Age**<br>F(2.12)=121.5<br>**Interaction**<br>F(2.12)=1.760	Sidak's Multiple Comparison Test	WT P7 vs JNK1 KO P7 = P<0.01<br><br>WT P15 vs JNK1 KO P15 = P<0.05<br><br>WT P90 vs JNK1 KO P90 = P<0.001
**Fig. 3B**	Two-way Anova (2-tailed)	WT P7 =3<br>JNK1 KO P7 =3<br>WT P15 =3<br>JNK1 KO P15 =3<br>WT P90 =3<br>JNK1 KO P90 =3	**Genotype**<br>P<0.01<br>**Age**<br>P<0.0001<br>**Interaction**<br>P<0.05	**Genotype**<br>F(1,12)=16.66<br>**Age**<br>F(2,12)=53.15<br>**Interaction**<br>F(2,12)=5.428	Sidak's Multiple Comparison Test	WT P7 vs JNK1 KO P7 = P<0.01<br><br>WT P15 vs JNK1 KO P15 = P<0.05<br><br>WT P90 vs JNK1 KO P90 = n.s.
**Fig. 3C**		WT P7 =3	**Genotype**	**Genotype**	Sidak's Multiple	

	Two-way Anova (2-tailed)	JNK1 KO P7 =3<br>WT P15 =3<br>JNK1 KO P15 =3<br>WT P90 =3<br>JNK1 KO P90 =3	P<0.001<br>**Age**<br>P<0.0001<br>**Interaction**<br>P<0.01	F(1,12)=24.95<br>**Age**<br>F(2,12)=80.16<br>**Interaction**<br>F(2,12)=7.315	Comparison Test	WT P7 vs JNK1 KO P7 = P<0.01<br><br>WT P15 vs JNK1 KO P15 = P<0.001<br><br>WT P90 vs JNK1 KO P90 = n.s.
**Fig. 3E**	Two-way Anova (2-tailed)	WT P7=3<br>JNK1 KO P7=3<br>WT P90=3<br>JNK1 KO P90=3	**Genotype**<br>P<0.01<br>**Age**<br>P<0.0001<br>**Interaction**<br>P<0.01	**Genotype**<br>F(1,8)=13.42<br>**Age**<br>F(1,8)=58.60<br>**Interaction**<br>F(1,8)=11.77	Sidak's Multiple Comparison Test	WT P7 vs JNK1 KO P7 = P<0.01<br>WT P90 vs JNK1 KO P90 = n.s.
**Fig. 3G**	Unpaired t test (2-tailed)	WT P7 =3<br>JNK1 KO P7=3	P=0.0054	t(4)=5.490		
**Fig. 3H**	Unpaired t test (2-tailed)	WT P15 =3<br>JNK1 KO P15=3	P=0.0301	t(4)=3.296		
**Fig. 3I**	Unpaired t test (2-tailed)	WT P90 =3<br>JNK1 KO P90=3	n.s.			
**Fig. 3K**	Two-way Anova (2-tailed)	WT P7 =3<br>JNK1 KO P7=3<br>WT P90=3<br>JNK1 KO P90=3	**Genotype**<br>n.s.<br>**Age**<br>n.s<br>**Interaction**<br>n.s	**Genotype**<br>F(1,8)=0.3061<br>**Age**<br>F(3,3)=0.4308<br>**Interaction**<br>F(1,8)=0.352	Sidak's Multiple Comparison Test	n.s
**Fig. 3L**	Two-way Anova (2-tailed)	WT P7 =3<br>JNK1 KO P7=3<br>WT P90=3<br>JNK1 KO P90=3	**Genotype**<br>P<0.01<br>**Age**<br>P<0.0001<br>**Interaction**<br>n.s	**Genotype**<br>F(1,8)=13.20<br>**Age**<br>F(1.8)=507.7<br>**Interaction**<br>F(1,8)=0.1180	Sidak's Multiple Comparison Test	WT P7 vs JNK1 KO P7 = P<0.05<br><br>WT P90 vs JNK1 KO P90 = n.s.
**Fig. 3M**	Unpaired t test (2-tailed)	WT P90 =3<br>JNK1 KO P90=3	n.s.			
**Fig. 3N**	Unpaired t test (2-tailed)	WT P90 =3<br>JNK1 KO P90=3	P=0.0225	t(4)=3.614		
**Fig. 3O**	Two-way Anova (2-tailed)	WT P90 =3<br>(orders=1-11)<br>JNK1 KO P90=3<br>(orders=1-6)	**Genotype**<br>P=0.0045<br>**Order**<br>P<0.0001<br>**Interaction**<br>n.s	**Genotype**<br>F(1,44)=8.957<br>**Order**<br>F(10,44)=81.14<br>**Interaction**<br>F(10,44)=0.7914	Sidak's Multiple Comparison Test	n.s
**Fig. 4B**	Chi square test	WT =3<br>JNK1 KO =3<br>WT: 2166 cells<br>JNK1 KO: 3889 cells	P<0.0001	$\chi 2(1)=121.0$		
**Fig. 4C**	Linear regression	WT =3<br>JNK1 KO =3	WT OPCs:<br>$R^2=0.1455$<br>Sy,x=11.51<br>Slope 95% confidence interval=<br>-0.1440 to -0.007284<br>WT slope ≠ 0<br>P=0.0312	**Slope**<br>F = 8.581<br>DFn = 1, DFd = 66<br>WT slope ≠ JNK1 KO slope<br>P<0.01		

			JNK1 KO OPCs:<br>R^2=0.1007<br>Sy,x=8.018<br>Slope 95% confidence interval=<br>-0.0006349 to 0.1228<br>JNK1 KO slope = 0<br>P>0.05			
**Fig. 4F**	Unpaired t test (2-tailed)	WT =3<br>JNK1 KO =3	n.s.			
**Fig. 4G**	Unpaired t test (2-tailed)	WT =3<br>JNK1 KO =3	P<0.05	t(4)=3.757		
**Fig. 4H**	Unpaired t test (2-tailed)	WT =3<br>JNK1 KO =3	P<0.01	t(4)=5.833		
**Fig. 4I**	Two-way Anova (2-tailed)	WT =3<br>(orders=1-14)<br>JNK1 KO =3<br>(orders=1-14)	**Genotype**<br>P<0.0001<br>**Order**<br>P<0.0001<br>**Interaction**<br>P<0.001	**Genotype**<br>F(1,56)=52.72<br>**Order**<br>F(13,56)=59.39<br>**Interaction**<br>F(13,56)=3.550	Sidak's Multiple Comparison Test	WT vs JNK1 KO:<br>Order 3 = P<0.0001<br>Order 4 = P<0.001<br>Order 5 = P<0.001
**Fig. 4J**	Two-way Anova (2-tailed)	WT =3<br>JNK1 KO =3	**Genotype**<br>P<0.0001<br>**Distance from soma**<br>P<0.0001<br>**Interaction**<br>P<0.0001	**Genotype**<br>F(1,44)=26.88<br>**Distance from soma**<br>F(10,44)=53.47<br>**Interaction**<br>F(10,44)=7.188	Sidak's Multiple Comparison Test	WT vs JNK1 KO:<br>5um = P<0.01<br>10um = P<0.0001<br>15um = P<0.0001<br>20um = P<0.05
**Fig. 5B**	Chi square test	CTRL =3<br>D-JNKI-1 =3<br>WT: 4081 cells<br>JNK1 KO: 4639 cells	P<0.0001	$\chi 2(1)$=57.22		
**Fig. 5D**	Two-way Anova (2-tailed)	CTRL =3<br>D-JNKI-1 =3	**Genotype**<br>P<0.05<br>**Distance from soma**<br>P<0.0001<br>**Interaction**<br>P<0.0001	**Genotype**<br>P<0.05<br>**Distance from soma**<br>P<0.0001<br>**Interaction**<br>P<0.0001	Sidak's Multiple Comparison Test	WT vs JNK1 KO:<br>10um = P<0.0001<br>20um = P<0.0001<br>30um = P<0.01
**Fig. 6B**	Chi square test	WT =4<br>JNK1 KO =4<br>WT: 87 cells<br>JNK1 KO: 95 cells	n.s.			
**Fig. 6C**	Two-way Anova (2-tailed)	WT =3<br>JNK1 KO =3	**Genotype**<br>n.s.<br>**Type**<br>P<0.0001<br>**Interaction**<br>n.s.	**Genotype**<br>F(1,12)=0.555<br>**Type**<br>F(1,12)=0.001<br>**Interaction**<br>F(1,12)=157.6	Sidak's Multiple Comparison Test	n.s.
**Fig. 6D**	Unpaired t test (2-tailed)	WT =3<br>JNK1 KO =3	P<0.05	t(4)=3.381		
**Fig. 6F**	Chi square test	CTRL =4<br>D-JNKI-1 =4<br>CTRL: 81 cells<br>D-JNKI-1: 96 cells	n.s.			

**Fig. 6G**	Two-way Anova (2-tailed)	CTRL =3<br>D-JNKI-1 =3	**Genotype**<br>n.s.<br>**Type**<br>P<0.001<br>**Interaction**<br>n.s.	**Genotype**<br>F(1,8)=0.1129<br>**Type**<br>F(1,8)=0.00018<br>**Interaction**<br>F(1,8)=3.169	Sidak's Multiple Comparison Test	n.s.
**Fig. 6H**	Unpaired t test (2-tailed)	WT =3<br>JNK1 KO =3	P<0.05	t(4)=3.557		
**Suppl. Fig. 1A**	Two-way Anova (2-tailed)	WT P15 suoragranular =3<br>JNK1 KO P15 supragranular P15 =3<br>WT P15 infragranular =3<br>JNK1 KO P15 infragranular P15 =3	**Genotype**<br>P<0.0001<br>**Layers**<br>P<0.01<br>**Interaction**<br>n.s.	**Genotype**<br>F(1,8)=53.95<br>**Layers**<br>F(1,8)=23.65<br>**Interaction**<br>F(1,8)=0.2038	Sidak's Multiple Comparison Test	WT P15 infragranular vs JNK1 KO P15 infragranular = P<0.05<br><br>WT P15 supragranular vs JNK1 KO P15 supragranular = P<0.05
**Suppl. Fig. 1B**	Two-way Anova (2-tailed)	WT P90 suoragranular =3<br>JNK1 KO P90 supragranular P15 =3<br>WT P90 infragranular =3<br>JNK1 KO P90 infragranular P15 =3	**Genotype**<br>P<0.001<br>**Layers**<br>P<0.0001<br>**Interaction**<br>n.s.	**Genotype**<br>F(1,8)=48.44<br>**Layers**<br>F(1,8)=77.16<br>**Interaction**<br>F(1,8)=4.58	Sidak's Multiple Comparison Test	WT P90 infragranular vs JNK1 KO P90 infragranular = P<0.05<br><br>WT P90 supragranular vs JNK1 KO P90 supragranular = P<0.001
**Suppl. Fig. 2A**	Unpaired t test (2-tailed)	WT P7 =3<br>JNK1 KO P7 =3<br>WT P15 =3<br>JNK1 KO P15 =3<br>WT P30 =3<br>JNK1 KO P30 =3	P7<br>SMI31 n.s.<br>MBP P<0.05<br>MBP/SMI31 P<0.05<br>MOG P<0.05<br>CNPase P<0.05<br><br>P15<br>SMI31 n.s.<br>MBP P<0.05<br>MBP/SMI31 P<0.05<br>MOG P<0.05<br>CNPase P<0.05<br><br>P30<br>SMI31 n.s.<br>MBP P<0.05<br>MBP/SMI31 P<0.01<br>MOG P<0.05<br>CNPase P<0.05	P7<br>SMI31 t(4)=1.217<br>MBP t(4)=3.018<br>MBP/SMI31 t(4)=4.595<br>MOG t(4)=3.349<br>CNPase t(4)=2.947<br><br>P15<br>SMI31 t(4)=0.6361<br>MBP t(4)=3.334<br>MBP/SMI31 t(4)=3.937<br>MOG t(4)=4.293<br>CNPase t(4)=3.025<br><br>P30<br>SMI31 t(4)=0.01895<br>MBP t(4)=3.258<br>MBP/SMI31 t(4)=7.205<br>MOG t(4)=3.228<br>CNPase t(4)=2.989		
**Suppl. Fig. 2B**	Unpaired t test (2-tailed)	WT P7 =3<br>JNK1 KO P7 =3<br>WT P15 =3<br>JNK1 KO P15 =3<br>WT P30 =3<br>JNK1 KO P30 =3	P7<br>SMI31 n.s.<br>MBP P<0.05<br>MBP/SMI31 P<0.05<br>MOG P<0.05<br>CNPase P<0.05<br><br>P15<br>SMI31 n.s.<br>MBP P<0.05<br>MBP/SMI31 P<0.05<br>MOG P<0.05<br>CNPase P<0.05<br><br>P30<br>SMI31 n.s.	P7<br>SMI31 t(4)=1.286<br>MBP t(4)=4.457<br>MBP/SMI31 t(4)=2.832<br>MOG t(4)=4.456<br>CNPase t(4)=3.321<br><br>P15<br>SMI31 t(4)=0.3421<br>MBP t(4)=3.425<br>MBP/SMI31 t(4)=3.401<br>MOG t(4)=3.200<br>CNPase t(4)=2.865<br><br>P30<br>SMI31 t(4)=0.2427		

			MBP P<0.05<br>MBP/SMI31 P<0.05<br>MOG P<0.05<br>CNPase P<0.01	MBP t(4)=3.063<br>MBP/SMI31 t(4)=2.959<br>MOG t(4)=3.016<br>CNPase t(4)=6.785		
**Suppl. Fig. 3A**	Two-way Anova (2-tailed)	WT P7 =3<br>JNK1 KO P7 =3<br>WT P15 =3<br>JNK1 KO P15 =3<br>WT P90 =3<br>JNK1 KO P90 =3	**Genotype**<br>n.s.<br>**Age**<br>P<0.0001<br>**Interaction**<br>n.s.	**Genotype**<br>F(1,12)=1.951<br>**Age**<br>F(2,12)= 91.67<br>**Interaction**<br>F(2,12)=2.108	Sidak's Multiple Comparison Test	n.s.
**Suppl. Fig. 3B**	Two-way Anova (2-tailed)	WT P7 =3<br>JNK1 KO P7 =3<br>WT P15 =3<br>JNK1 KO P15 =3<br>WT P90 =3<br>JNK1 KO P90 =3	**Genotype**<br>n.s.<br>**Age**<br>P<0.0001<br>**Interaction**<br>n.s.	**Genotype**<br>F(1,12)=1.165<br>**Age**<br>F(2,12)=43.06<br>**Interaction**<br>F(2,12)=1.381	Sidak's Multiple Comparison Test	n.s.
**Suppl. Fig. 3C**	Two-way Anova (2-tailed)	WT P7 infragranular =3<br>JNK1 KO P7 infragranular =3<br>WT P15 infragranular =3<br>JNK1 KO P15 infragranular =3<br>WT P7 supragranular =3<br>JNK1 KO P7 supragranular =3<br>WT P15 supragranular =3<br>JNK1 KO P15 supragranular =3	**Genotype**<br>P<0.001<br>**Age**<br>P<0.0001<br>**Interaction**<br>n.s.	**Genotype**<br>F(1,16)=18.36<br>**Age**<br>F(3.16)=15.13<br>**Interaction**<br>F(3.16)=0.6775.	Sidak's Multiple Comparison Test	WT P7 infragranular vs WT P7 supragranular = n.s.<br><br>JNK1 KO P7 infragranular vs JNK1 KO P7 supragranular = n.s.<br><br>WT P15 infragranular vs WT P15 supragranular = n.s.<br><br>JNK1 KO P15 infragranular vs JNK1 KO P15 supragranular = n.s.
**Suppl. Fig. 3D**	Two-way Anova (2-tailed)	WT P7=3<br>JNK1 KO P7=3<br>WT P90=3<br>JNK1 KO P90=3	**Genotype**<br>P<0.05<br>**Age**<br>P<0.0001<br>**Interaction**<br>P<0.05	**Genotype**<br>F(1,8)=10.8<br>**Age**<br>F(1,8)=175.60<br>**Interaction**<br>F(1,8)=7.46	Sidak's Multiple Comparison Test	WT P7 vs JNK1 KO P7 = P<0.01<br>WT P90 vs JNK1 KO P90 = n.s.

**Suppl. Fig. 4A**	Chi square test	WT =4 coverslips/2 exp<br>JNK1 KO =4 coverslips/2 exp<br>WT: 1143 cells<br>JNK1 KO: 2125 cells	P<0.001	$\chi^2(1)=12.64$		
**Suppl. Fig. 4B**	Linear regression	WT =4 coverslips/2 exp<br>JNK1 KO =4 coverslips/2 exp	WT OPCs:<br>R^2=0.1486<br>Sy,x=1.939<br>Slope 95% confidence interval=<br>-0.1293 to -0.006151<br>WT slope ≠ 0<br>P=0.0322<br><br>JNK1 KO OPCs:<br>R^2=0.3985<br>Sy,x=2.678<br>Slope 95% confidence interval=<br>-0.1036 to -0.03851<br>JNK1 KO slope ≠ 0<br>P=0.0001	**Slope**<br>F = 0.007319<br>DFn = 1, DFd = 59<br>n.s.<br><br>**Intercepts**<br>F = 56.54.<br>DFn = 1, DFd = 60<br>P<0.0001		
**Suppl. Fig. 4C**	Unpaired t test (2-tailed)	WT =4<br>JNK1 KO =4	P<0.0001	t(6)=17.73		
**Suppl. Fig. 4D**	Unpaired t test (2-tailed)	WT =4<br>JNK1 KO =4	n.s.	t(6)=0.1402		
**Suppl. Fig. 4E**	Unpaired t test (2-tailed)	WT =4<br>JNK1 KO =4	n.s.	t(6)=1.378		
